# Pseudorabies virus UL38 attenuates the cGAS-STING signaling pathway by recruiting Tollip to promote STING for autophagy degradation

**DOI:** 10.1186/s12985-024-02379-x

**Published:** 2024-05-08

**Authors:** Zhenfang Yan, Jingying Xie, Zhengyang Hou, Yaxin Zhang, Jiayu Yue, Xiangbo Zhang, Lei Chen, Yanmei Yang, Xiangrong Li, Huixia Li, Ruofei Feng

**Affiliations:** 1 Key Laboratory of Biotechnology and Bioengineering of State Ethnic Affairs Commission, Biomedical Research Center, Northwest Minzu University, No.1 Xibeixincun, Lanzhou, 730030 China; 2 College of Life science and Engineering, Northwest Minzu University, Lanzhou, 730030 China; 3 Gansu Tech Innovation Center of Animal Cell, Biomedical Research Center, Northwest Minzu University, Lanzhou, 730030 China

**Keywords:** UL38 protein, type I IFN signaling pathway, STING, Selective autophagy

## Abstract

Natural immunity is the first defense line of the host immune system, which plays a significant role in combating foreign pathogenic microorganisms. The IFN-β (interferon-beta) signaling pathway, being a typical example of innate immunity, plays a vital function. This study aimed to elucidate the function of pseudorabies virus (PRV) UL38 protein (unique long region 38) in suppressing the activation of the IFN-β signaling pathway. The findings from our study indicate that the PRV UL38 protein effectively hampers the activation of IFN-β by poly (dA: dT) (poly(deoxyadenylic-deoxythymidylic)) and 2’3’-cGAMP (2’-3’-cyclic GMP-AMP). Furthermore, UL38 exhibits spatial co-localization with STING (stimulator of interferon genes) and effectively hinders STING dimerization. Subsequently, STING was downgraded to suppress the production of IFN-β and ISGs (interferon stimulated genes). Immunoprecipitation analysis revealed that the interaction between UL38 and STING, which subsequently initiated the degradation of STING via selective autophagy mediated by TOLLIP (toll interacting protein). To summarize, this research elucidates the function of UL38 in counteracting the cGAS (cGAMP synthase)-STING-induced IFN-β pathway. The PRV UL38 protein may attenuate the activation of IFN-β as a means of regulating the virus’s persistence in the host.

**Supplementary Information:**

The online version contains supplementary material available at 10.1186/s12985-024-02379-x.

## Introduction

Pseudorabies, also referred to as Aujeszky’s disease, is a viral infection caused by the pseudorabies virus (PRV). This disease is a severe feverish infection that is known for causing disorders in the nervous system and high death rates among piglets. It also leads to respiratory problems and weight loss of finishing pigs, as well as reduced fertility in female pigs [1]. This disease has resulted in significant financial losses for the global breeding industry. PRV belongs to the *Varicellovirus* genus, *α-Herpesvirinae* subfamily within the *Herpesviridae* family. The genome of PRV is a DNA molecule that is double-stranded and has a length of around 150 kilobases. It consists of two distinct regions, namely the unique long (UL) region and the unique short (US) region. There are 70 open reading frames in the coding region that encoded 70–100 viral proteins, including 11 glycoproteins [2, 3].

The UL38 protein is a common nucleocapsid protein that participates in the formation of viral particles and has a crucial function in the replication of viruses [4]. Like other herpesviruses, the PRV nucleocapsid protein has a triplex structure composed of a VP19C protein (Tri1, UL38 gene) and two VP23 proteins (Tri2, UL18 gene) attached to the lower part of the capsid through the N-terminal end of Tri1 [5, 6]. Numerous researches have indicated that UL38 plays a key role in the life cycle of HCMV infection. Additionally, the HCMV UL38 protein is accountable for various elements of HCMV-induced metabolic activation. UL38 is both essential and adequate in stimulating glycolysis and initiating specific amino acid catabolism [7]. The UL38 protein contains a nuclear export signal that is found specifically at amino acid positions 342–351 [8, 9]. UL38 encodes a protein that can shuttle between the nucleus and cytoplasm in a chromosomal region maintenance 1 (CRM1)-dependent manner involving Ran GTP hydrolysis. This makes UL38 the first herpesvirus capsid protein with nucleoplasmic shuttling properties and adds it to the ranks of HSV-1 nucleoplasmic shuttling proteins [10, 11]. However, the current understanding of UL38’s role in controlling the replication and pathogenesis of PRV has not been fully elucidated.

The antiviral innate immunity response in host cells is initiated by pattern-recognition receptors (PRRs) that detect invading viruses. DNA sensors, including TLR9 (toll like receptor 9), IFI16 (interferon gamma inducible protein 16), AIM2 (absent in melanoma 2), ZBP1/DAI (Z-DNA binding protein 1), DDX41 (DEAD-box helicases 41), and cGAS (cyclic GMP-AMP synthase), play crucial roles in this process [12–16]. Typically, the cGAS-STING pathway becomes active upon binding viral or cellular DNA in the cytoplasm of cells, resulting in the generation of the secondary messenger compound known as 2’3’-cyclic GMP-AMP (cGAMP). Subsequently, cGAMP acts as a molecule that triggers the activation of STING1 (a protein involved in the interferon response called cGAMP interactor 1) and the downstream TBK1 (a kinase called TANK binding kinase 1) and the transcription factor IRF3 (known as interferon regulatory factor 3) pathways [17]. Therefore, cGAS plays a crucial role in initiating type I IFN production and controlling the innate immune responses against DNA viruses [18, 19].

Since the DNA sensing pathway activated by innate immunity plays a crucial role in regulating PRV infection, PRV proteins have developed intricate strategies to counteract the innate immune response. Multiple studies have suggested that PRV UL13 hinders the production of IFN-β by directly affecting IRF3 in a manner dependent on its kinase activity [20–22]. PRV UL24 efficiently inhibited the production of IFN through cGAS-STING pathway by interacting with interferon regulatory factor 7 (IRF7) and degrading its expression [23]. Furthermore, PRV gE plays a role in inhibiting cGAS-STING induced IFN production by breaking down CBP (CREB-binding protein) [24]. Our previous research also discovered that the PRV US3 protein has the ability to hinder the production of IFN through the cGAS-STING pathway by interacting with IRF3 and causing degradation of its expression [25]. According to a recent investigation, the interaction between PRV UL24 and ICP0 (infected cell protein 0) proteins with p65 leads to the degradation of p65 through proteasomal pathway, thereby inhibiting TNF-α (tumor necrosis factor alpha) induced NF-κB (nuclear factor-kappa B) activation [26, 27].

Several studies have shown that PRV has evolved multiple strategies to evade the host innate immune response, but the underlying mechanisms for immune evasion are still not fully understood. In the current research, the role of PRV UL38 in regulating the cGAS-STING mediated IFN-β induction is further explored. We discovered that the UL38 protein suppresses cGAS-STING induced antiviral signaling by breaking down STING via autophagy pathway. UL38 mechanistically induced the degradation of STING through selective autophagy mediated by TOLLIP. The results of our study uncovered a significant mechanism that PRV uses to evade the immune response of the host against viral infections. Our findings revealed an important mechanism underlying evasion of the host antiviral immune response by PRV.

## Materials and methods

### Cells and viruses

PK15, HEK293, and BHK-21 cells were cultured in DMEM (Bailing, Lanzhou, China) at 37 °C in a 5% CO_2_ incubator, supplemented with 10% NBS (new bovine serum). The Bartha-K61 strain was cultured in BHK-21 cells and kept in our laboratory.

### Antibodies and reagents

Sangon Biotech (Shanghai, China) provided the following antibodies: rabbit polyclonal antibody against the FLAG tag (D191041), rabbit polyclonal antibody against the HA tag (D110004), rabbit polyclonal antibody against cGAS (D163570), HRP-conjugated Goat Anti-Rabbit IgG (D110058) and HRP-conjugated Goat Anti-Mouse IgG (D110087). The Polyclonal antibody for IRF3 (11312-1-AP) was acquired from Proteintech in Wuhan, China. The antibodies for phospho-IRF3 (Ser386) (37829 S), STING (13647 S), TBK1 (3013 S), and Myc-Tag (9B11) Mouse mAb (2276 S) were purchased from Cell Signaling Technology. The GAPDH Mouse Monoclonal Antibody (AF5009) and the β-actin Mouse Monoclonal Antibody (AA128) were purchased from Beyotime Biotechnology (Shanghai, China).

TransStart® Top Green qPCR SuperMix (+ Dye II) was bought from Transgen (Beijing, China). Invitrogen was the supplier of Lipofectamine 3000. The proteasome inhibitor MG132 (S1748) and the apoptosis inhibitor Z-VAD-FMK (C1202) were bought from Beyotime. InvivoGen provided the lysosomal pathway inhibitor chloroquine (CQ) (tlrl-chq), 2’3’-cGAMP (tlrl-nacga23-02), and poly (dA: dT) (tlrl-patc).

### Plasmids

Plasmids encoding Flag-tagged TBK1, IRF3(5D), HA-tagged cGAS, STING and Myc-tagged UL38 were constructed in-house. All plasmids were verified by sequencing. Selective autophagy receptors plasmids Myc-Tollip, Myc-NDP52, Myc-p62 and Myc-OPTN were purchased from Public Protein/Plasmid Library. Plasmids encoding C-terminal-STING and N-terminal-STING was provided by Dr. Shasha Li (College of Life science and Engineering, Northwest Minzu University, Lanzhou).

### Western blotting

Harvested cells were used to prepare whole-cell extracts by using lysis buffer RIPA (Solarbio, Beijing, China). Protein samples were analyzed using 10% SDS-PAGE, and then transferred onto polyvinylidene fluoride (PVDF) membranes (Millipore Corp, Bedford, MA, United States). The PVDF membranes were incubated with specific primary and HRP-conjugated secondary antibodies. GAPDH or β-actin was used as a reference for loading. ECL Blotting Substrates (Bio-Rad, CA, United States) were used to identify the proteins.

### Co-immunoprecipitation assay

Cells were collected using a lysis buffer supplemented with a phosphatase inhibitor. They were then subjected to a 12 h incubation at 4 ℃ with either anti-Myc, anti-FLAG, or anti-HA antibody. Next, each lysate was supplemented with 20 µL of Protein G agarose slurry obtained from Beyotime. Following a 4 h incubation at 4 ℃, the lysates underwent centrifugation at a speed of 2500 rpm for 5 min. The beads were collected and washed 5 times with ice-cold PBS. The precipitates were mixed with SDS buffer and boiled for 5 min at 95 ℃. After centrifugation at 6000 rpm for 1 min, the supernatant was collected and used for western blot analysis.

### RNA isolation and quantitative real-time PCR (RT-qPCR)

RT-qPCR was used to measure the expression levels of mRNAs encoding IFN-β, ISG15, and MX1. According to the guidelines provided by the manufacturer, PK15 cells were used to extract total RNA using a Total RNA Isolation Reagent (Transgene, Beijing, China). Afterwards, the RNA samples were converted into cDNA through reverse transcription. Subsequently, a two-step RT-qPCR was carried out using a SYBR^®^ Green assay with an Applied Biosystems Master Mix kit in an ABI 7500 Real-Time PCR system. β-actin was used as the internal control. The relative fold change was determined using the threshold cycle (2^∆−Ct^) method for all data. The RT-qPCR primers can be found in Table 1.

### Virus infection

Cells were infected with viruses at the indicated multiplicity of infection (MOI). After adsorption for 2 h, the monolayers were overlaid with DMEM supplemental with 3% NBS and incubated at 37 °C. For viral titer determination, samples were harvested at 12, 24 or 36 h post infection and viruses, released by three cycles of freezing and thawing, were titrated on BHK-21 cells. For western blot assay, the cell lysates were harvested at indicated timepoints for examination.

### Virus titer

BHK-21 cells in 96-well plates were infected with 10-fold serial dilutions of PRV samples. The plates were placed in the cell incubator and left to incubate for one hour. Next, the culture medium was replaced with a new batch of DMEM. The cells were placed in an incubator for an additional 72 h at a temperature of 37 ℃. We observed CPE and calculated PRV titers using the Reed-Muench method.

### Statistical analysis

A one-way ANOVA was utilized to compare the measurements. A Student’s t-test was utilized to calculate statistical significance comparisons. Graph bars represent the average ± standard deviation (SD) of a minimum of three separate trials, unless specified otherwise. Statistically significant differences are indicated by asterisks (*** *p* < 0.001, ** *p* < 0.01 and * *p* < 0.05).

**Table 1:** Primers utilized for RT-qPCR

Primers	Sequences (5’-3’)
sIFN-β-qF	TCCACCACAGCTCTTTCCAT
sIFN-β-qR	CTGGAATTGTGGTGGTTGCA
sISG15-qF	AGGGAACTGAAGGTGAAGATG
sISG15-qR	CAGACGCTGCTGGAAGG
sMX1-qF	AGAGGCAGCGGAATTGTGAC
sMX1-qR	TTTCCACCTGCGAAGCATCT
sβ-actin-qF	CAAGGACCTCTACGCCAACAC
sβ-actin-qR	TGGAGGCGCGATGATCTT

## Results

### The transcription of IFN-β, ISG15, and MX1 is inhibited by the PRV-encoded viral protein UL38

**Fig. 1:**
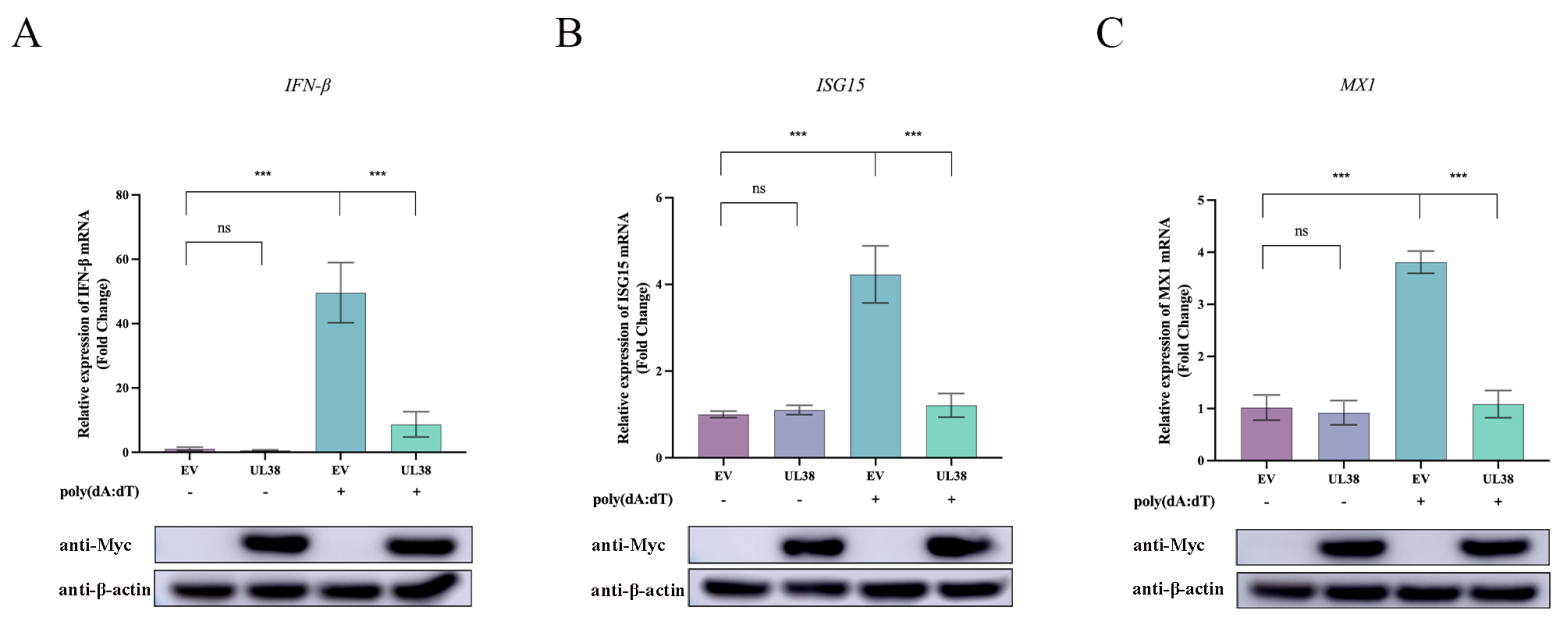
UL38 suppresses poly(dA: dT)-mediated mRNA levels of IFN-β, ISG15 and MX1. PK15 cells were cultured overnight in 6-well plates, and Myc-tagged UL38 expression plasmid (1 µg) was transfected for 24 h. Then cells were stimulated with poly(I: C) (1 µg/mL) for another 12 h. Collected cells for cellular RNA extraction. 1 µg RNA was transcripted into cDNA for *IFN-β* (**A**), *ISG15* (**B**) and *MX1* (**C**) mRNA detection. Data were expressed as mean ± SD from three independent experiments. Comparisons between groups were performed by Student’s t test (unpaired). *** *p* < 0.001

**Fig. 2:**
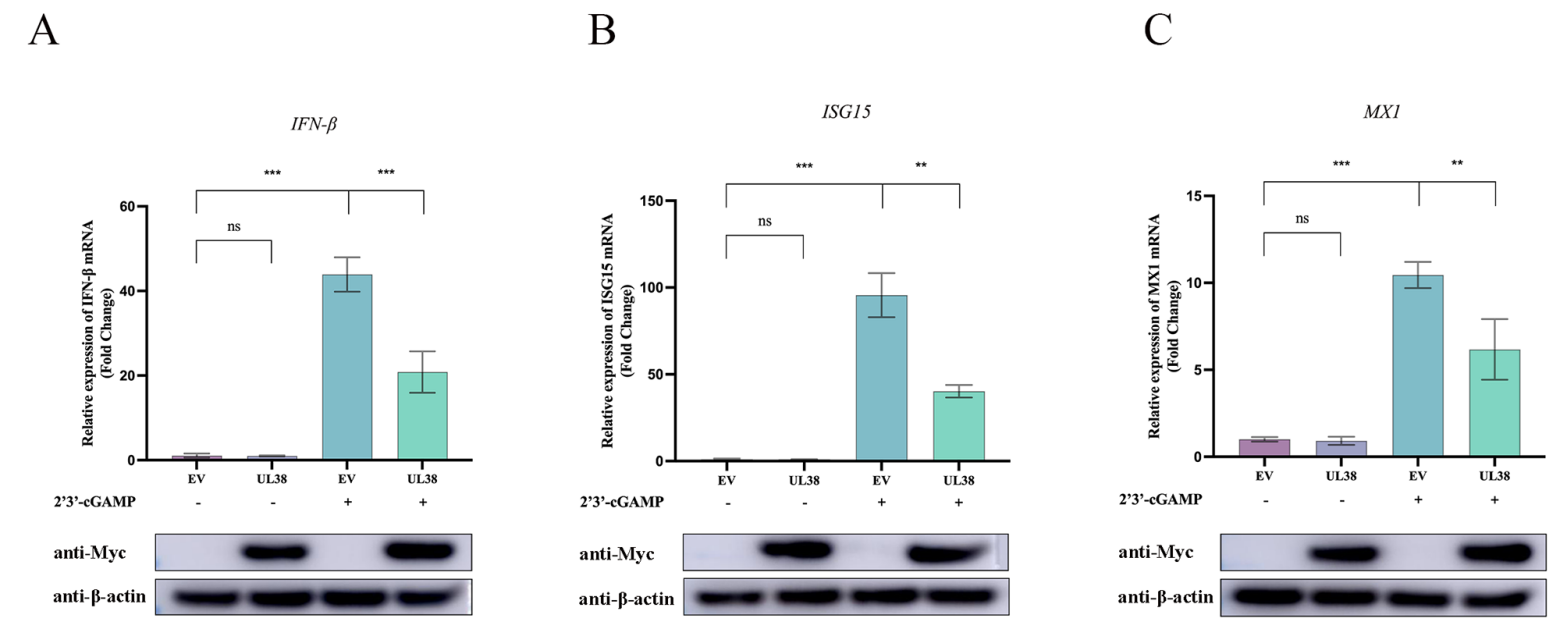
UL38 suppresses 2’3’-cGAMP-triggered mRNA levels of IFN-β, ISG15 and MX1. PK15 cells were cultured overnight in 6-well plates, and Myc-tagged UL38 expression plasmid (1 µg) was transfected for 24 h. Then cells were transfected and stimulated with 2’3’-cGAMP (2 mg/mL) for another 12 h. Collected cells for cellular RNA extraction. 1 µg RNA was transcripted into cDNA for *IFN-β* (**A**), *ISG15* (**B**) and *MX1* (**C**) mRNA detection. Data were expressed as mean ± SD from three independent experiments. Comparisons between groups were performed by Student’s t test (unpaired). ** *p* < 0.01, *** *p* < 0.001

PRV, being a virus with double-stranded DNA, has employed various strategies to hinder the signaling pathways of DNA sensors in order to promote its replication [28]. Our previous studies found that PRV US3, ICP0, UL41 significantly inhibited the IFN-β signaling pathway [25, 27]. Based on these studies, we have great interest in whether the capsid protein of PRV can mediate the immune escape of PRV. Thus, we postulated that UL38 of PRV could potentially contribute to overcoming the limitation of innate immunity mediated by DNA sensors. As shown in Fig. 1, PRV UL38 was found to suppress poly(dA: dT)-induced IFN-β transcription. The transcription levels of MX1 and ISG15 were also suppressed. Furthermore, 2’3’-cGAMP acts as a secondary signal to trigger the innate immune system by attaching to STING (stimulator of IFN genes) and subsequently stimulating the TBK1-IRF3-dependent synthesis of IFN-β [29]. During the stimulation of 2’3’-cGAMP, we subsequently observed the mRNA expression of IFN-β, MX1, and ISG15. Comparable results were achieved in comparison to the poly(dA: dT) treatment group. The mRNA transcript levels of the factors mentioned above were significantly reduced by the ectopic expression of UL38, as illustrated in Fig. 2. The findings indicated that UL38 suppressed the production of antiviral components triggered by poly(dA: dT) and 2’3’-cGAMP.

### UL38 could potentially target STING

**Fig. 3:**
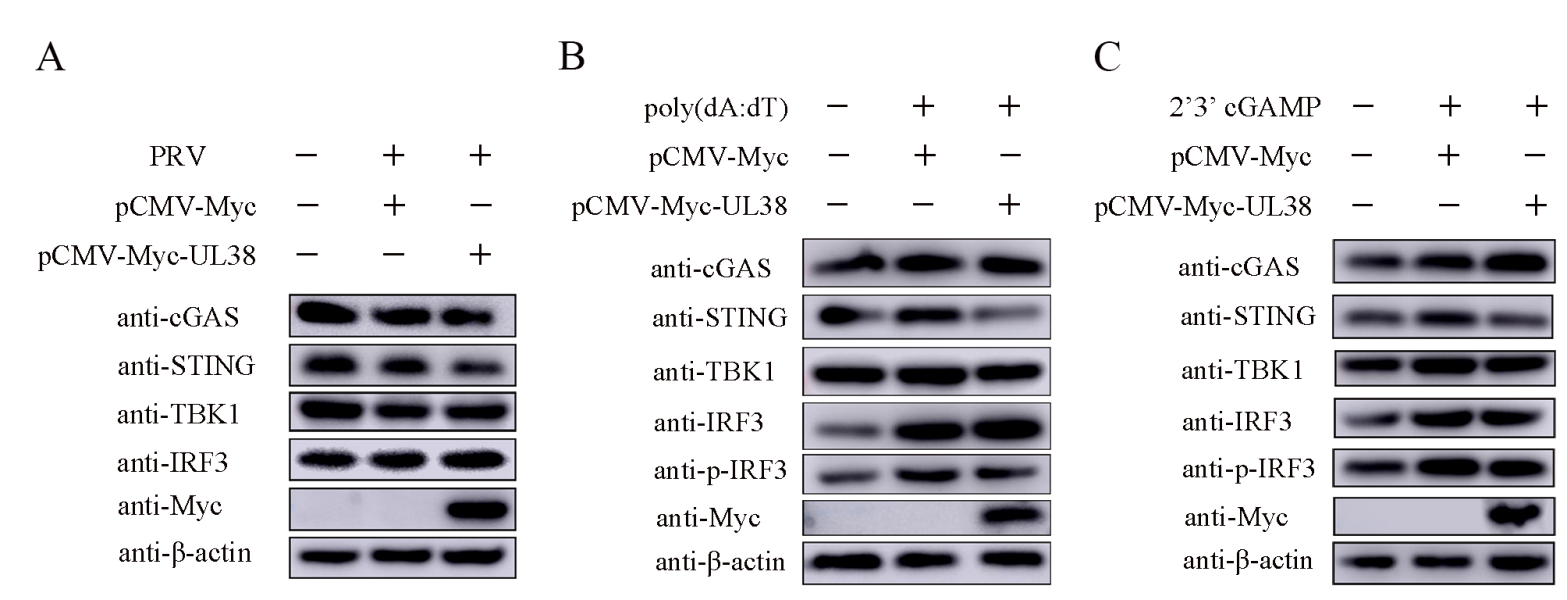
UL38 promotes decreasing of STING. PK15 cells were transfected with pCMV-Myc (1 µg) or Myc-UL38 (1 µg) for 24 h, then cells were stimulated with 1 MOI PRV (**A**), poly(dA: dT) (1 µg/mL) (**B**) or 2’3’-cGAMP (2 mg/mL) (**C**) respectively for another 12 h. Cells were collected for western blotting analysis. The expression of cGAS, STING, TBK1, IRF3 and phosphorylated IRF3 were detected. For all experiments, β-actin serves as a loading control

The cGAS-STING pathway has a crucial function in the innate immune response to viral infections [17]. Therefore, we hypothesized that UL38 could potentially inhibit the cGAS-STING mediated innate immune response. In order to confirm the impact of UL38 on the cGAS-STING signaling pathway, an analysis was conducted on the expression of cGAS, STING, TBK1, and IRF3 in PK15 cells expressing UL38. Notably, it was observed that the expression of STING decreased upon transfection cells with PRV UL38 (Fig. 3), suggesting that PRV UL38 hinders the innate immunity mediated by cGAS-STING by promoting the degradation of STING.

### The autophagy-lysosome pathway is responsible for the degradation of STING by PRV UL38

**Fig. 4:**
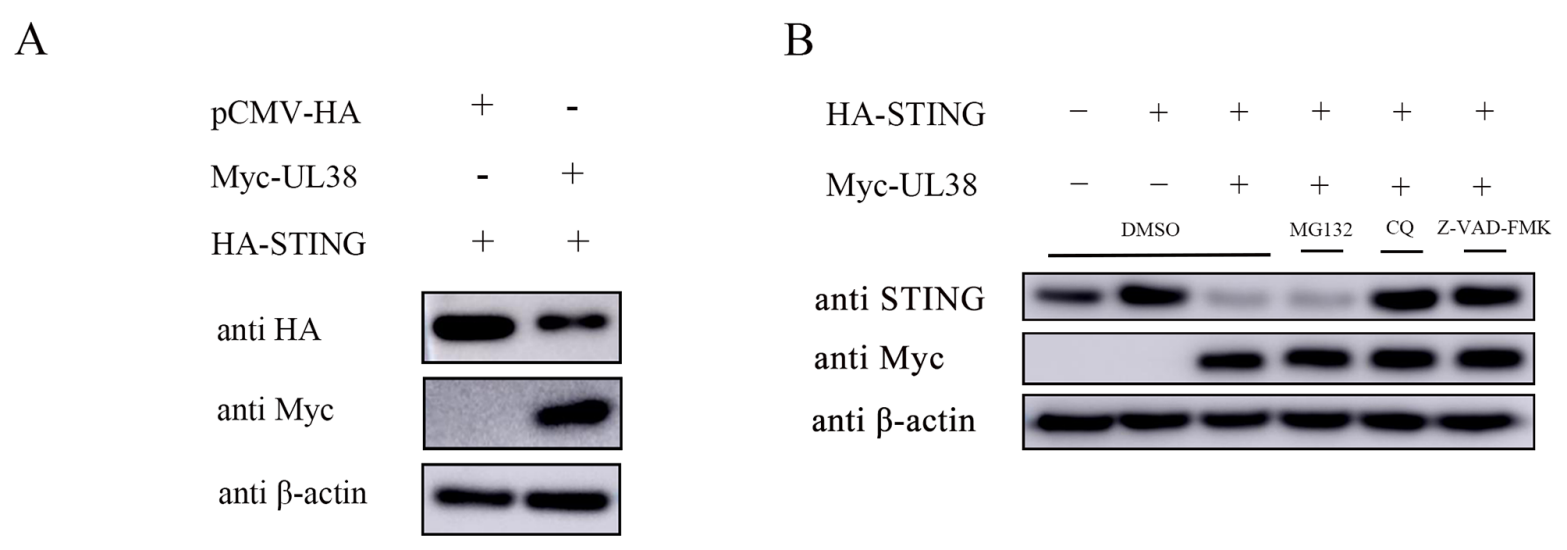
UL38 degrades STING through the autophagy-lysosome pathway. (**A**) PK15 cells were cotransfected with HA-STING (500 ng) and Myc-UL38 (1 µg) for 24 h, then cells were collected for wetsern blotting analysis. β-actin served as a loading control. (**B**) PK15 cells were transfected with Myc-UL38 (1 µg) or pCMV-Myc empety vector (EV) (1 µg) for 24 h, then treated with proteasomal inhibitor MG132 (7.5 µM), caspase inhibitor Z-VAD-FMK (50 µM) or the autophagy-lysosome inhibitor CQ (50 µM) for 12 h. DMSO treated cells served as vehicle control. Then cells were collected and immunoblotting for STING and Myc. β-actin served as a loading control

In order to further analyze the impact of UL38 on the expression of STING, we assessed the protein level of STING in cells that were transfected with HA-STING plasmid, either with UL38 or with an empty vector. According to the data presented in Fig. 4A, the cells co-transfected with UL38 exhibited a reduction in STING expression, whereas no change was observed in the cells transfected with the vector plasmid.

In eukaryotic cells, protein degradation is regulated by three primary mechanisms: the caspase pathway, the ubiquitin-proteasome pathway, and the autophagy-lysosome pathway [30–32]. In order to identify the pathway responsible for the degradation of STING, PK15 cells were co-transfected with STING and UL38, and subsequently treated with DMSO, Z-VAD-FMK, MG132, or chloroquine (CQ). The expression of STING was restored in cells treated with CQ and Z-VAD-FMK, as depicted in Fig. 4B, whereas it was not observed in cells treated with DMSO or MG132. These findings suggested that UL38 reduces the expression of STING by utilizing the autophagy-lysosome pathway.

### UL38 interacts with STING

**Fig. 5:**
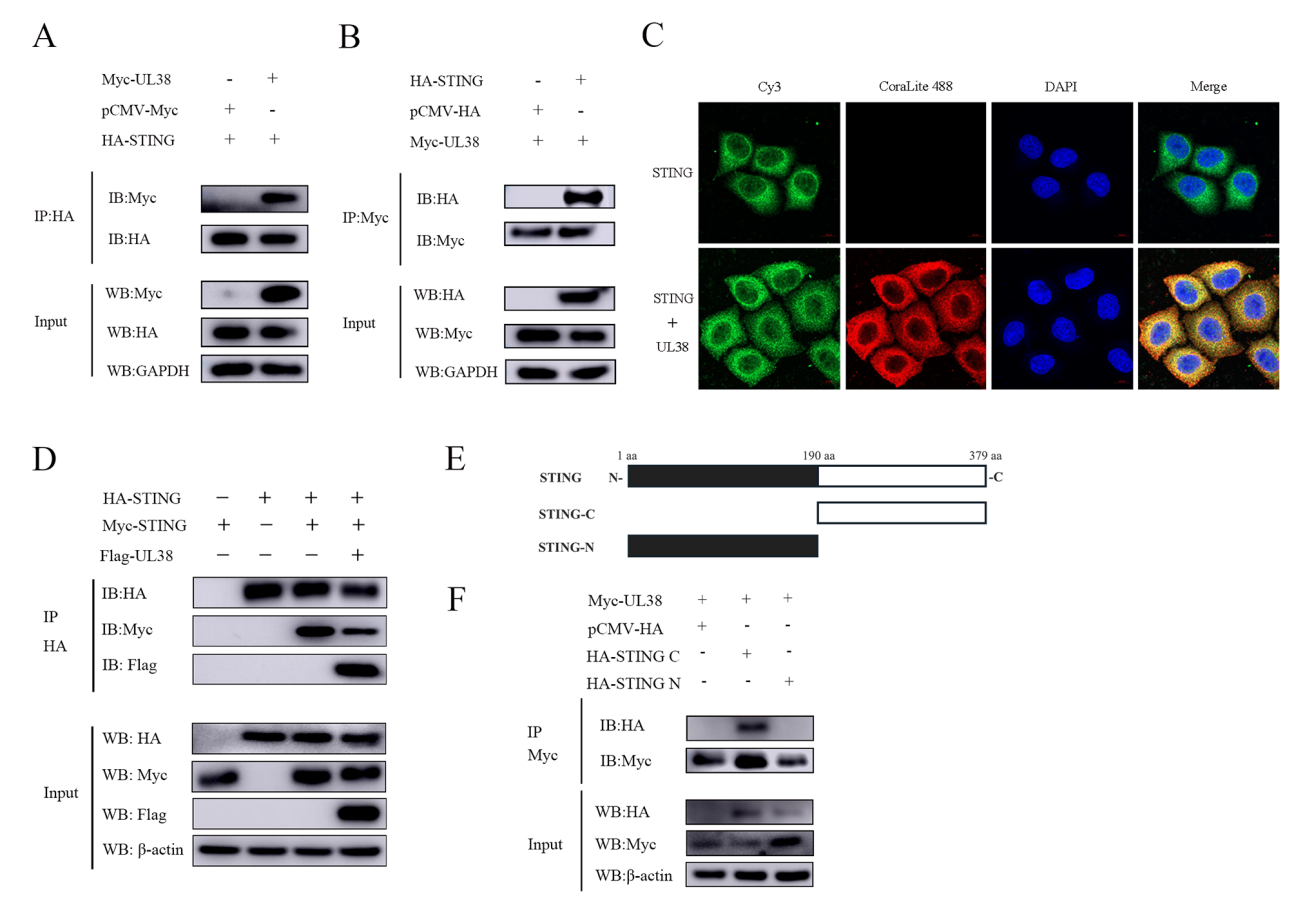
UL38 interacts with STING (**A**) PK15 cells were co-transfected with empty vector (500 ng) or Myc-UL38 (500 ng) plasmids and HA-STING (500 ng) plasmids for 30 h. The cells were then lysed and immunoprecipitated with an anti-HA antibody. The whole-cell lysates (input) and immunoprecipitation (IP) complexes were analyzed by western blotting using an anti-HA, anti-Myc or anti-GAPDH antibody. (**B**) PK15 cells were co-transfected with empty vector (500 ng) or HA-STING (500 ng) plasmids and Myc-UL38 (500 ng) plasmids for 30 h. The cells were then lysed and immunoprecipitated with an anti-Myc antibody. The whole-cell lysates (input) and immunoprecipitation (IP) complexes were analyzed by western blotting using an anti-Myc, anti-HA or anti-GAPDH antibody. (**C**) PK15 cells were transfected with empty vector (500 ng) or Myc-UL38 (500 ng). At 24 h, cells were stained with anti-Myc (red) and anti-STING (green) subjected to analysis by confocal microscopy. Nuclei were stained with DAPI (blue). Scale bars, 10 μm. (**D**) PK15 cells were co-transfected with empty vector (500 ng) or FLAG-UL38 (500 ng) plasmids and HA-STING (500 ng) along with Myc-STING (500 ng) plasmids for 30 h. The cells were then lysed and immunoprecipitated with an anti-HA antibody. The whole-cell lysates (input) and immunoprecipitation (IP) complexes were analyzed by western blotting using an anti-HA, anti-Myc, anti-FLAG or anti-β-actin antibody. (**E**) Schematic of the truncated STING. STING is truncated into two segments on average and labeled as STING-C and STING-N, respectively. (**F**) PK15 cells were co-transfected with Myc-UL38 (500 ng) and pCMV-HA empty vector (500 ng) or Myc-UL38 (500 ng) and HA-STING C (500 ng) plasmids or HA-STING N (500 ng) plasmid for 30 h. The cells were then lysed and immunoprecipitated with an anti-Myc antibody. The whole-cell lysates (input) and immunoprecipitation (IP) complexes were analyzed by western blotting using an anti-Myc, anti-HA or anti-β-actin antibody

To determine if the degradation events resulted from the molecules’ interactions, we conducted coimmunoprecipitation (Co-IP) to verify the interaction between UL38 and STING. The interaction between HA-tagged STING and Myc-tagged UL38 was discovered (Fig. 5A and B). STING plays a crucial role as an adapter molecule in the DNA-sensing pathway, exhibiting significant antiviral properties [33]. For a more thorough examination, STING was chosen for additional study. The co-occurrence of STING and UL38 was verified by confocal microscopy (Fig. 5C). After analyzing the aforementioned findings, we firmly believe that UL38 specifically focuses on STING, engaging in an interaction that leads to the degradation of its functions. Consequently, this process restricts the activation of type I IFN.

In order to examine the potential involvement of UL38 in STING signaling, we conducted co-transfection experiments using plasmids labeled differently from STING. The purpose was to confirm the impact of UL38 on the occurrence of STING dimerization. The findings from Fig. 5D demonstrated a significant inhibitory effect of UL38 on STING dimerization. In order to investigate the specific regions of STING that directly interacted with UL38, we created a range of shortened mutants, including N-terminal-STING-1-190aa and C-terminal-STING-191-379aa (Fig. 5E). PK15 cells successfully expressed both plasmids, and Co-IP analysis further validated the interaction between UL38 and C-STING (Fig. 5F). This suggests that the C-terminal domain of STING plays a crucial role in the degradation of STING mediated by UL38.

### The degradation of STING induced by UL38 is attributed to autophagy facilitated by TOLLIP

**Fig. 6:**
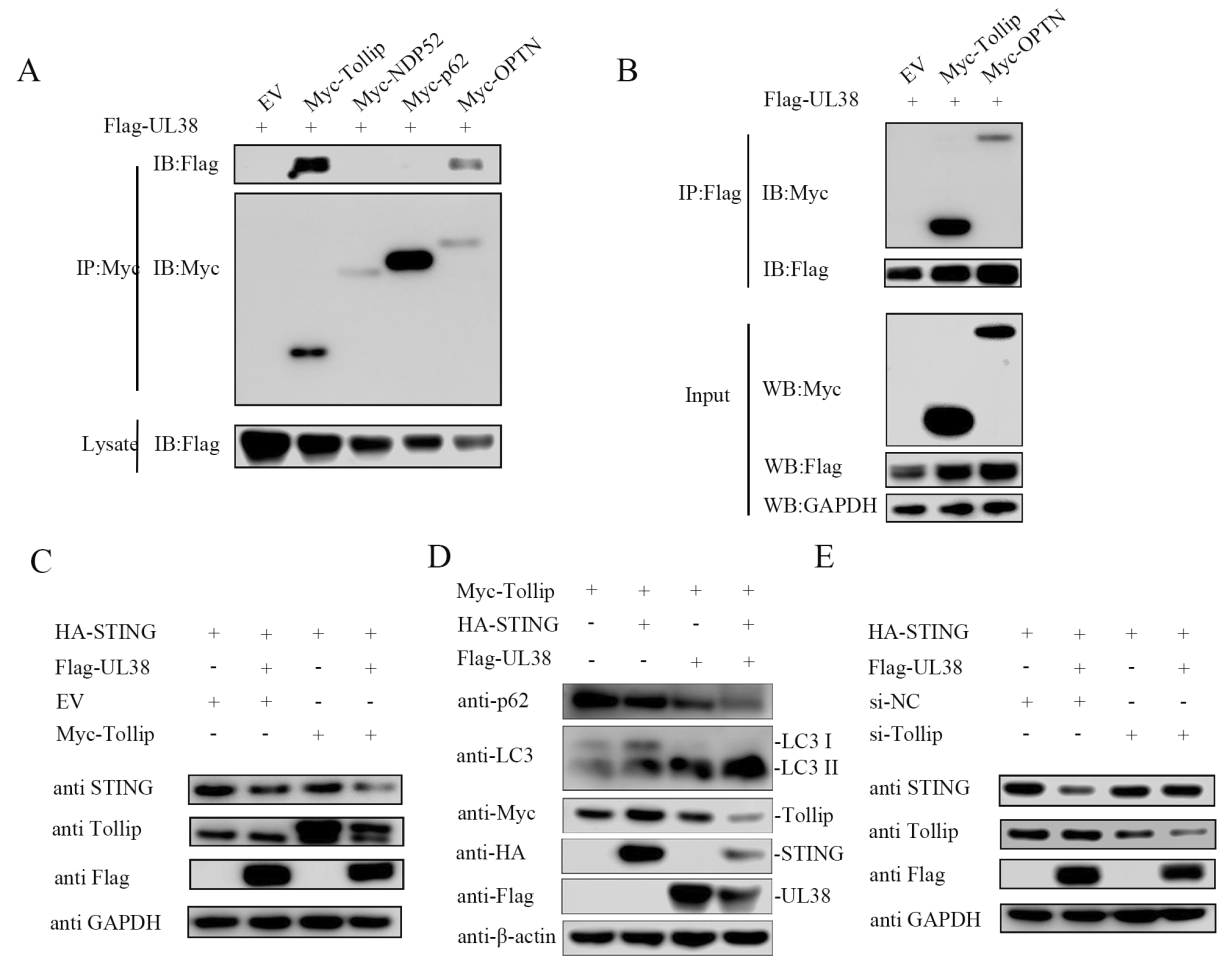
UL38 recruits Tollip to promote STING autophagic degradation. (**A**) PK15 cells were cotransfected with Myc-tagged selective autophagy receptor expression plasmids (Tollip, NDP52, p62 and OPTN) (500 ng) and Flag-UL38 (1 µg) plasmid for 30 h. The cells were then lysed and immunoprecipitated with an anti-Myc antibody. The whole-cell lysates (input) and immunoprecipitation (IP) complexes were analyzed by western blotting using an anti-Myc or anti-FLAG antibody. (**B**) PK15 cells were cotransfected with Flag-UL38 (1 µg) plasmid and Myc-tagged selective autophagy receptor expression plasmids (Tollip and OPTN) (500 ng) for 30 h. The cells were then lysed and immunoprecipitated with an anti-Flag antibody. The whole-cell lysates (input) and immunoprecipitation (IP) complexes were analyzed by western blotting using an anti-Flag, anti-Myc or anti-GAPDH antibody. (**C**) PK15 cells were cotransfected with Myc-Tollip (500 ng), HA-STING (500 ng) and Flag-UL38 (1 µg) plasmid for 24 h. The cells were then collected and lysed for STING, Tollip and Flag tagged UL38 detection. GAPDH served as a loading control. (**D**) Myc-Tollip transfected PK15 cells were feeded in 6-well plate, then transfected with HA-STING (500 ng), FLAG-UL38 (1 µg) or both HA-STING (500 ng) and FLAG-UL38 (1 µg) plasmid. Cells were collected and lysed for LC3, HA tagged STING, Myc tagged Tollip and Flag tagged UL38 detection. β-actin served as a loading control. (**E**) PK15 cells were transfected with siRNA targeting Tollip, or negative control siNC. Then cells were cotransfected with HA-STING (500 ng) and Flag-UL38 (1 µg) plasmid for 24 h. The cells were then collected and lysed for STING, Tollip and Flag tagged UL38 detection. GAPDH served as a loading control

After witnessing a consistent and robust suppression of STING by UL38, our curiosity was piqued regarding the mechanism by which UL38 facilitated the degradation of STING via the autophagy pathway. For the degradation of STING, it is necessary to have particular cargo receptors that connect invasion proteins and autophagosomes during selective autophagy [34]. Our hypothesis suggests that these specific autophagy receptors might be responsible for delivering STING to autophagosomes. The Co-IP experiments demonstrated that UL38 had interactions with Tollip and OPTN (optineurin), excluding CALCOCO2/NDP52 (calcium binding and coiled-coil domain 2) or SQSTM1/p62 (sequestosome 1) as shown in Fig. 6A and B. Tollip was selected for further examination. The degradation of STING induced by UL38 was more apparent when UL38 and Tollip were co-transfected with STING, as shown in Fig. 6C. As UL38 degaraded STING through autophagy pathway, LC3 and p62 were detected. As shown in Fig. 6D, in Tollip, STING and UL38 cotransfected group, p62 and LC3-I expression were decreased and LC3-II expression increased, indicating complete autophagy occurs. In contrast, the degradation of STING induced by UL38 was restored after interference with Tollip expression (Fig. 6E). The findings indicated that increased levels of UL38 enhanced the degradation of STING through Tollip.

### UL38 promotes PRV infection in PK15 cells

**Fig. 7:**
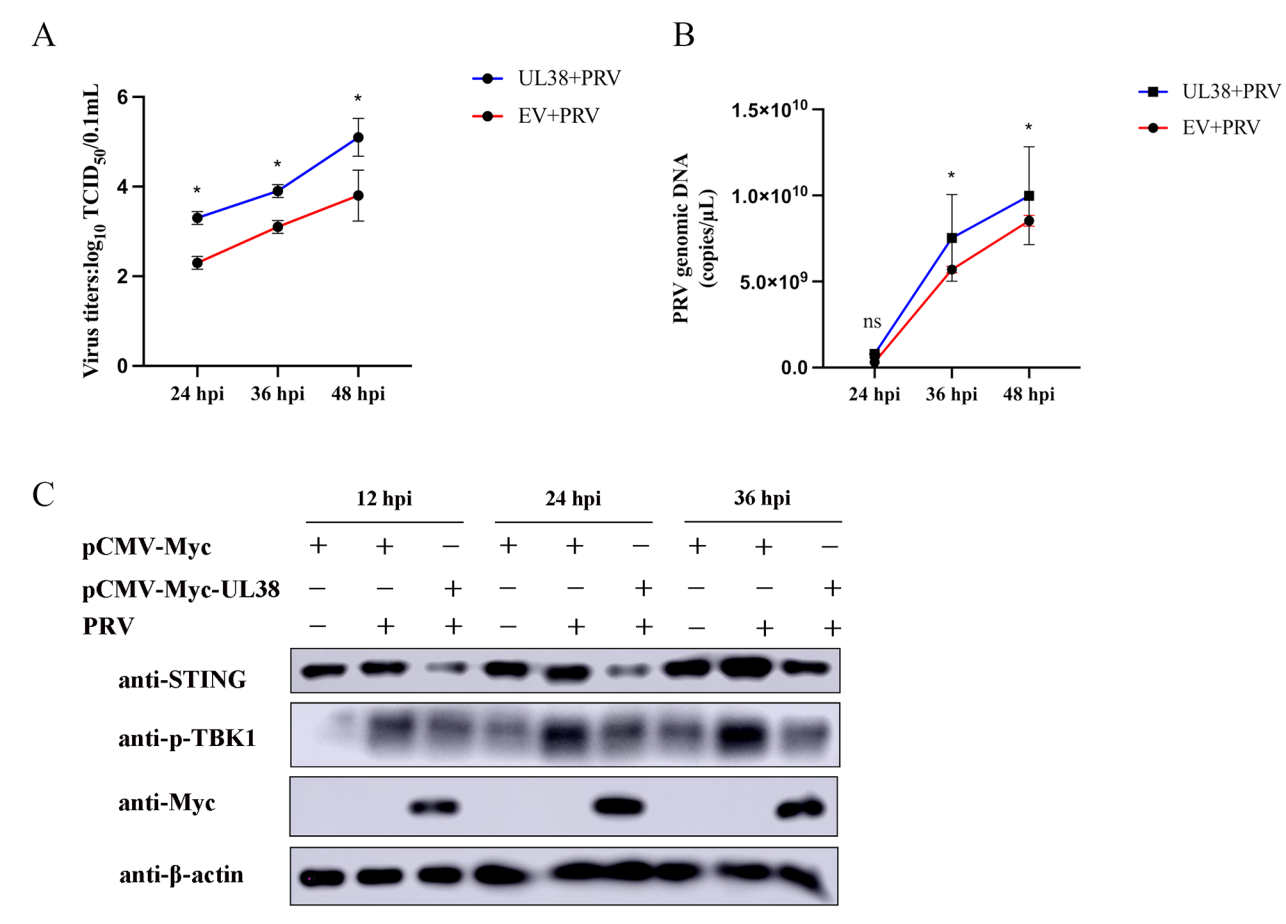
UL38 promotes PRV replication in PK15 cells. PK15 cells were transfected with pCMV-Myc (1 µg) or Myc-UL38 (1 µg) infected with PRV (0.01 MOI) for 24 h, 36 h and 48 h. The supernatant was used to measure viral titers by TCID_50_ assay (**A**). The viral DNA was assessed by RT-qPCR (**B**). *p < 0.05. (**C**) STING blot (12, 24, and 36 h post infection) after UL38 + PRV and EV + PRV infection was detected by western blotting. β-actin served as a loading control

In order to examine the function of viral protein UL38 in PRV infection, PK15 cells were transfected with pCMV-Myc-UL38 plasmid for investigation. Following a 24-hour period, cells were infected with PRV to assess the impact of UL38 on the multiplication of PRV. The replication of PRV was detected using TCID_50_. According to Fig. 7A, the findings indicated a notable rise in the virus titers of PRV in the UL38 group compared to the EV group. Comparable findings were acquired for the detection of virus copy quantity through RT-qPCR (Fig. 7B). In addition to detecting viral titers, we also examined the protein expression levels of STING and found that PRV infection effectively inhibited the expression of STING and p-TBK1 (Fig. 7C). Similar results that again confirmed that PRV infection was associated with a significant reduction in the expression of STING. The findings indicate that the UL38 viral protein of PRV greatly enhances the replication of PRV in PK15 cells and viral protein UL38 is involved in the degradation of STING to antagonize the innate immune response.

## Discussion

**Fig. 8:**
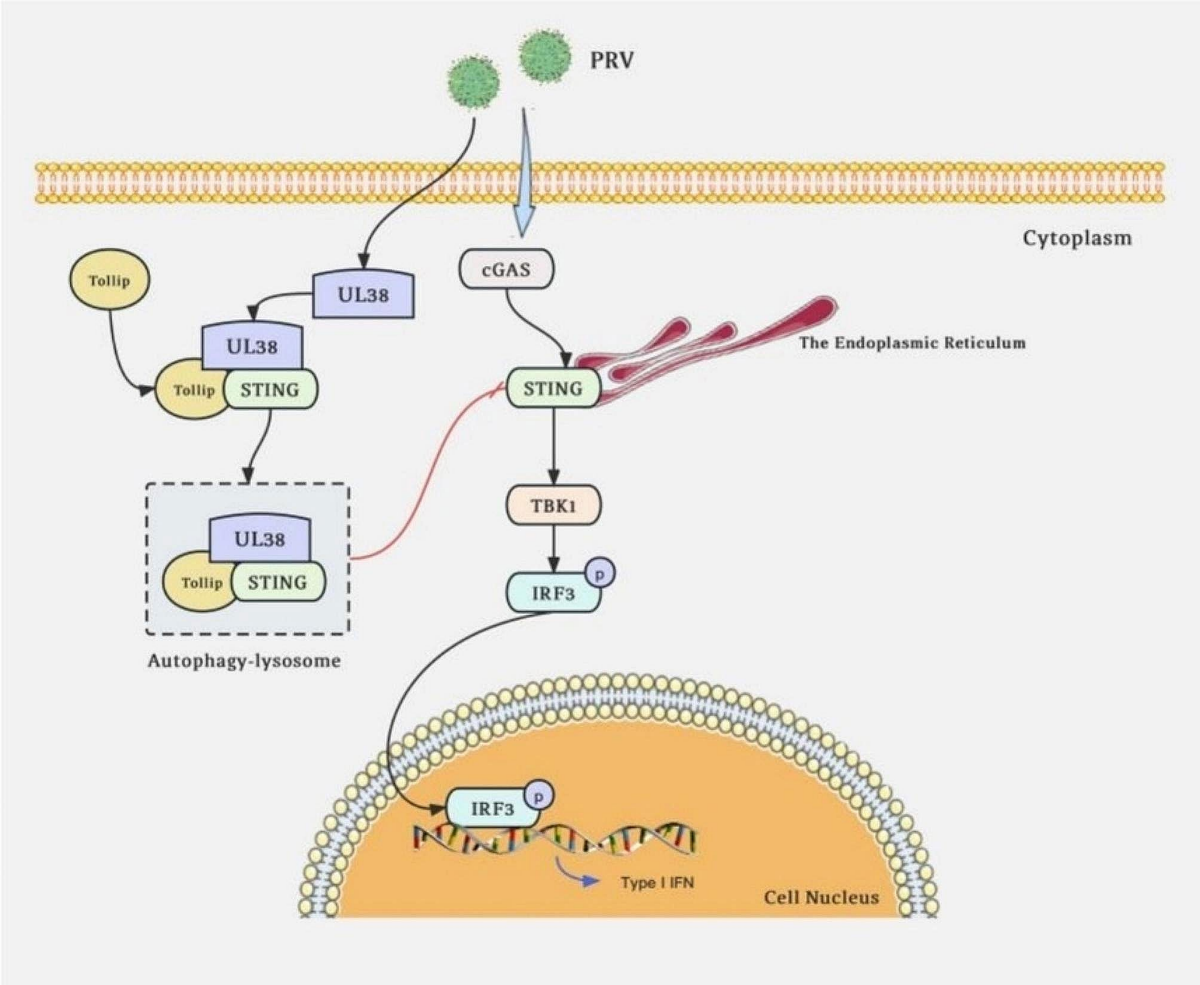
Schematic representation of PRV UL38 targeting STING to inhibit IFN-I activity. UL38 interacts with and degrades STING via the autophagy pathway by inducing the selective autophagy receptor Tollip, followed by the inhibition of type I IFN response

PRV triggers innate immune responses through the cytoplasmic DNA sensor known as cGAS. Conversely, PRV usually tries to undermine the immune response of the host in order to enhance their own life cycle. This study presents a model in which the UL38 capsid protein in PRV counteracts the innate signaling pathway mediated by cGAS-STING (Fig. 8). In summary, we demonstrated that the UL38 protein prompts the degradation of STING as a means to counter the immune response against the host’s antiviral defense. Afterwards, the UL38 protein engages with the specific autophagic receptor TOLLIP to identify STING, ultimately merging with the lysosome to degrade STING. Consequently, UL38 hinders the activation of STING, resulting in the inhibition of type I interferon and an increased efficiency in viral replication.

The UL38 protein is a common nucleocapsid protein that participates in the formation of viral particles and has a crucial function in the replication of viruses [4]. Comparing the amino acid sequence of PRV UL38 with that of other herpesviruses showed notable similarity in structure [35]. UL38 protein in Human Cytomegalovirus (HCMV) is essential for various metabolic changes caused by HCMV, such as the stimulation of glucose and glutamine usage, as well as the secretion of lactate. The metabolic reprogramming function of UL38 relies on its interaction with TSC2 (tuberous sclerosis 2), a tumor suppressor that hinders mTOR (mammalian target of rapamycin) signaling [7]. Furthermore, HCMV UL38 additionally inhibits apoptosis, preventing early demise of host cells in order to enhance virus replication effectively [9]. The research on the activation of transcription by the promoter of herpes simplex virus type 1 (HSV-1) UL38 has been extensively studied [36, 37]. The genes UL37 and UL38 are located next to each other on the viral DNA, but they are transcribed from different strands. The UL37 gene codes for a protein with 1,123 amino acids whose function remains unknown, whereas the UL38 protein, consisting of 465 amino acids, plays a role in the assembly of the capsid [38]. In this study, we have verified that UL38 plays a crucial role in facilitating efficient virus replication during PRV infection. Through TOLLIP-mediated selective autophagy, UL38 exerts a significant influence on the suppression of cGAS-STING signaling. This study demonstrated that PRV UL38 have the ability to cause degradation of STING. Further exploration in the future is necessary to determine if the role of UL38 in STING degradation is preserved in other alpha-herpesvirus counterparts.

Despite the fact that PRV have developed various strategies to counteract the host’s innate immune response, there are still very few methods to counteract the innate immune mechanism, and the roles of certain proteins remain poorly understood. The research discovered that UL38 hindered the activation of IFN-β through cGAS-STING pathway. Additionally, UL38 inhibited the expression of IFN-β and ISGs. Further research indicated that UL38 had interactions with STING and potentially played a role in controlling the autophagic breakdown of STING, thereby suppressing the IFN-I pathway. To summarize, this data uncovers the role of UL38 and its inhibitory mechanism on the innate antiviral response, potentially offering a new theoretical foundation for the development of novel vaccines.

STING, an indispensable downstream adaptor protein in cGAS, plays a crucial role in antiviral defense [39]. Serving as a foundation for a signaling cascade reliant on TBK1 transmission, STING facilitates the phosphorylation and activation of IRF3, thereby triggering an immune response against viruses by enhancing the transcription of IFN-I [40]. Through targeting STING for degradation, the UL38 protein of PRV was found to inhibit the host antiviral immune response in this investigation. There are many pathways for protein degradation, such as proteasome pathway, autophagy pathway and apoptosis pathway. Our research revealed that the autophagy pathway is responsible for UL38-mediated degradation of STING.

Autophagy can be classified into non-selective and selective autophagy based on the substrate it targets for degradation [41]. Autophagy induced by STING is the primary function from an evolutionary standpoint, occurring prior to the activation of interferon function. This process is not reliant on TBK1-IRF3 and the conventional autophagy signaling pathway [42]. Additionally, a different study demonstrated that STING undergoes oligomerization instead of phosphorylation, a process necessary for FMDV-induced autophagy [43]. The maintenance of tissue immune homeostasis is achieved through competitive regulation of resting STING by Tollip and IRE1a-lysosome. Through the autophagy-lysosome pathway, it attaches to UNC93B1, a protein located in the endoplasmic reticulum, and facilitates its breakdown [44]. According to our findings, UL38 facilitates the autophagic degradation of STING by causing the evasion of host defense through the activation of the selective autophagy receptor Tollip. Nonetheless, additional research is required to ascertain if there is a disturbance in the equilibrium between IRE1a-lysosome and Tollip.

## Conclusions

This study aimed to investigate the function of UL38 in inhibiting the innate immune response through regulation of the cGAS-STING-mediated pathway for type I IFN signaling. Certainly, UL38 facilitates the autophagic breakdown of STING through Tollip and plays a crucial role in enabling PRV to evade the immune response. The result presents a novel approach for how PRV UL38 can manipulate the molecular immune response of innate immunity to facilitate infection.

### Electronic supplementary material

Below is the link to the electronic supplementary material.

Supplementary Material 1

## Data Availability

No datasets were generated or analysed during the current study.
