# Supplementary material for: Pseudorabies virus UL38 attenuates the cGAS-STING signaling pathway by recruiting Tollip to promote STING for autophagy degradation
Source: Virol J. 2024 May 8;21:107. doi: 10.1186/s12985-024-02379-x (PMC11080157; doi:10.1186/s12985-024-02379-x)
Supplement: Supplementary file 1 — Supplementary Material 1 [file 12985_2024_2379_MOESM1_ESM.docx]

Figure 1

A

| EV | UL38 | EV+poly(dA:dT) | UL38+poly(dA:dT) |
| --- | --- | --- | --- |
| 1 | 0.614 | 49.02572632 | 8.084821701 |
| 0.600517213 | 0.482 | 40.57760239 | 5.087427139 |
| 1.665231228 | 0.782 | 59.23271942 | 12.84821033 |


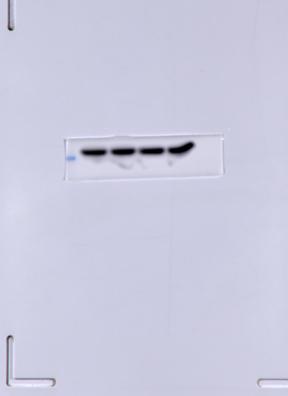

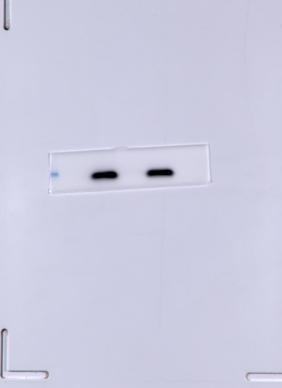


beta-actin Myc-UL38

B

| EV | UL38 | EV+poly(dA:dT) | UL38+poly(dA:dT) |
| --- | --- | --- | --- |
| 1 | 1.198393656 | 4.197138786 | 1.191825151 |
| 0.926989853 | 1.128236519 | 3.589514256 | 0.949944258 |
| 1.078760505 | 0.983374629 | 4.907620907 | 1.495295167 |


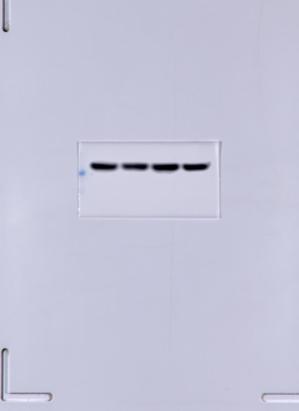

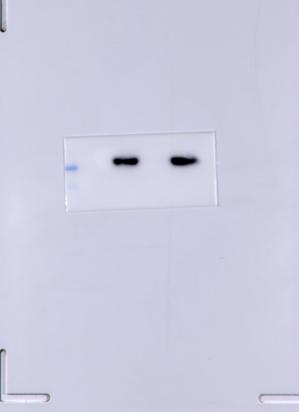


beta-actin Myc-UL38

C

| EV | UL38 | EV+poly(dA:dT) | UL38+poly(dA:dT) |
| --- | --- | --- | --- |
| 1 | 0.9 | 3.765422821 | 1.065696955 |
| 0.787317038 | 0.697 | 3.62227726 | 0.836307526 |
| 1.270136356 | 1.162 | 4.041047573 | 1.358005166 |


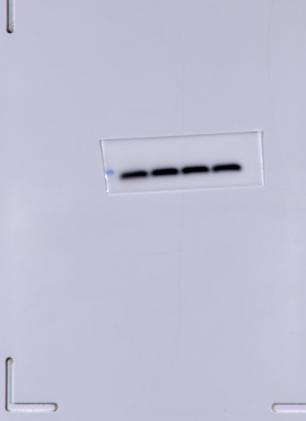

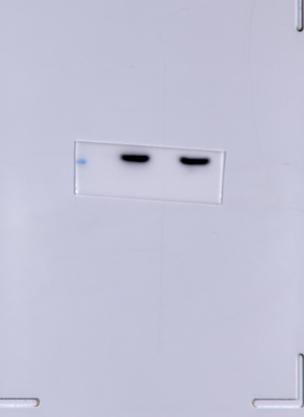


beta-actin Myc-UL38

Figure 2

A

| EV | UL38 | EV+2’3’cGAMP | UL38+2’3’cGAMP |
| --- | --- | --- | --- |
| 1 | 1 | 41.5644989 | 25.70705795 |
| 6.22430742e-001 | 0.89 | 48.57738686 | 20.897892 |
| 1.606604457 | 1.124 | 41.5644989 | 15.897892 |


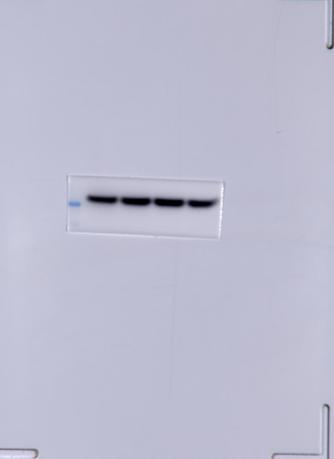

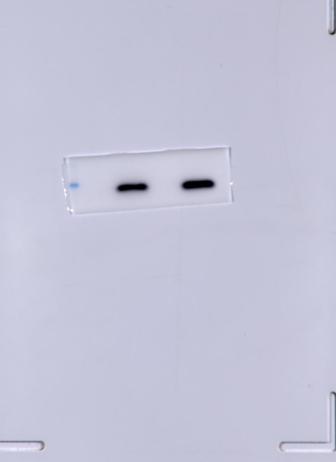


beta-actin Myc-UL38

B

| EV | UL38 | EV+2’3’cGAMP | UL38+2’3’cGAMP |
| --- | --- | --- | --- |
| 1 | 0.956 | 83.02648926 | 39.91811371 |
| 1.72829705e-001 | 0.804 | 82.82200241 | 34.17707062 |
| 1.682189703 | 1.311 | 120.9779205 | 46.62353516 |


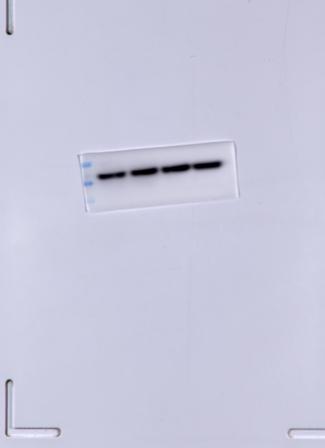

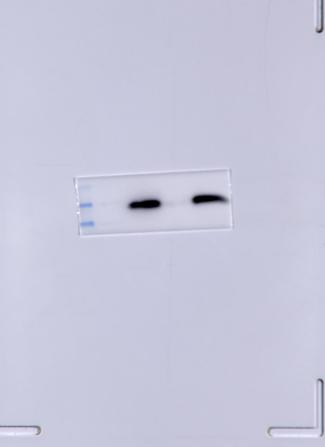


beta-actin Myc-UL38

C

| EV | UL38 | EV+2’3’cGAMP | UL38+2’3’cGAMP |
| --- | --- | --- | --- |
| 1 | 0.9 | 10.4317255 | 7.997097015 |
| 8.77582252e-001 | 0.697 | 9.705586433 | 4.52648592 |
| 1.1394943 | 1.162 | 11.21219254 | 5.997097015 |


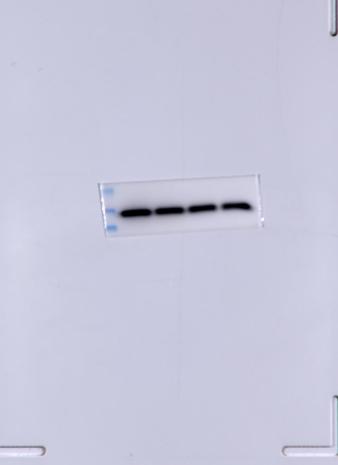

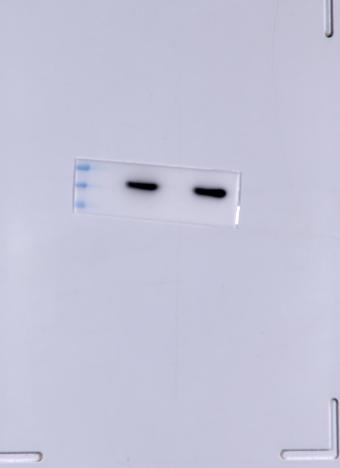


beta-actin Myc-UL38

Figure 3

A


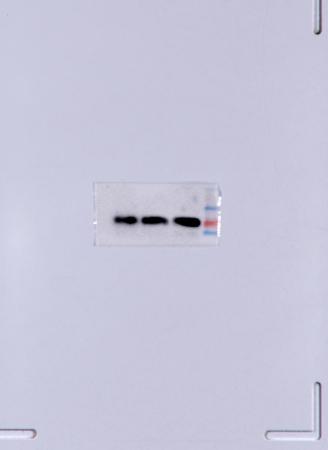

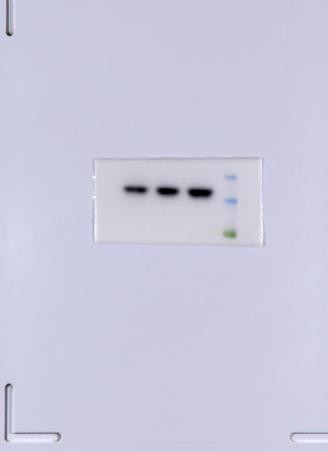

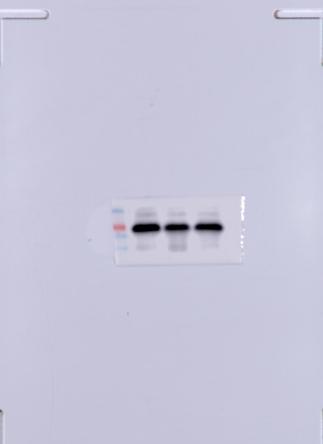


cGAS STING TBK1


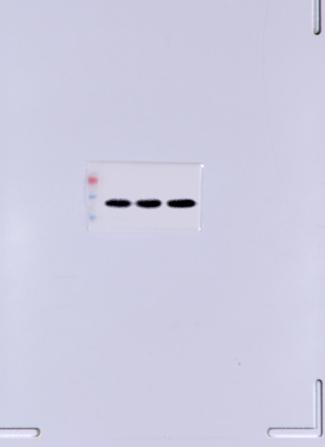

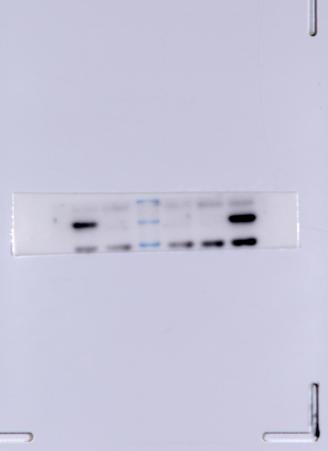

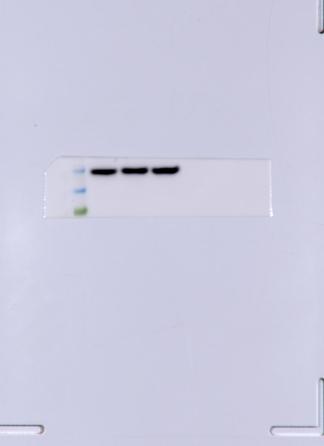


IRF3 Myc-UL38 beta-actin

B


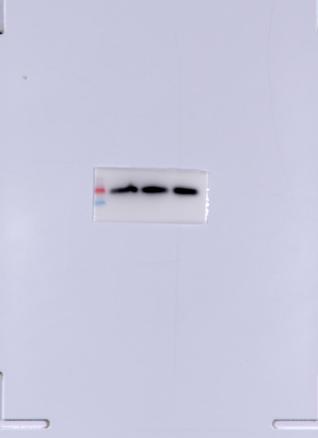

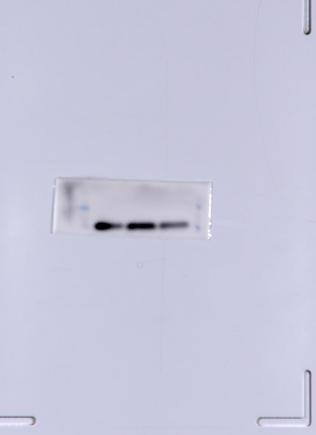

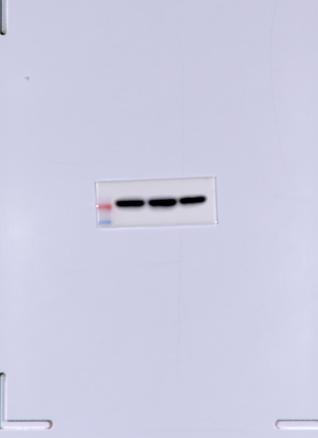


cGAS STING TBK1


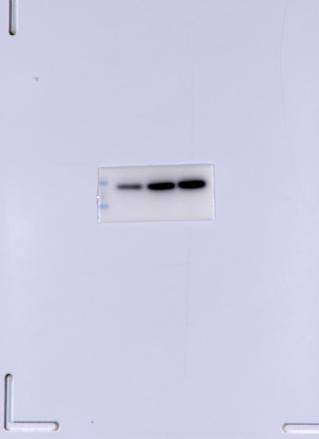

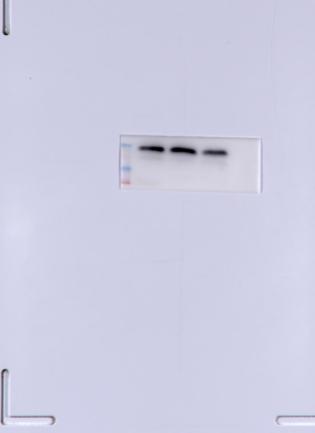

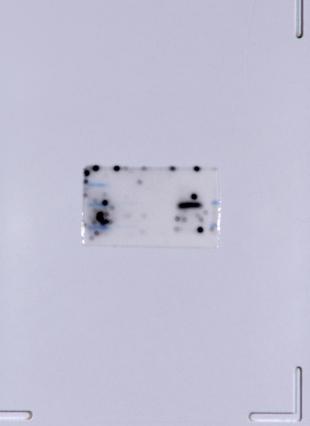


IRF3 p-IRF3 Myc-UL38


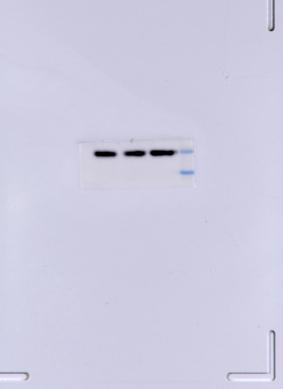


beta-actin

C


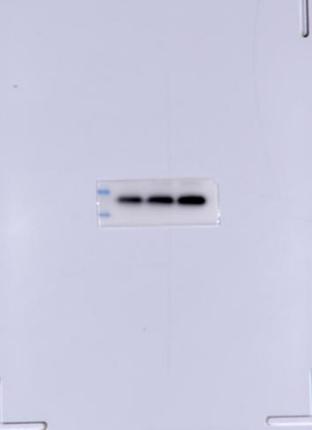

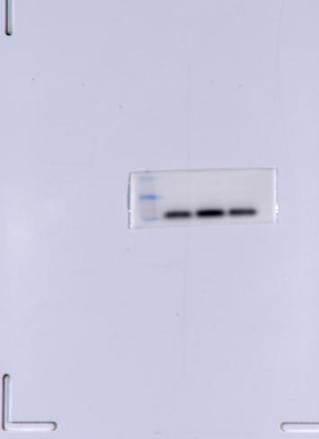

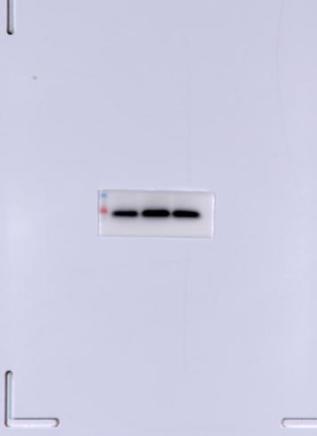


cGAS STING TBK1


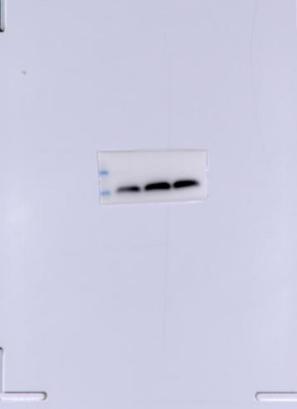

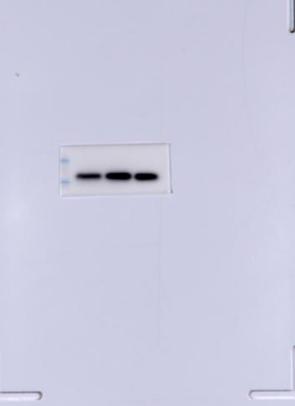

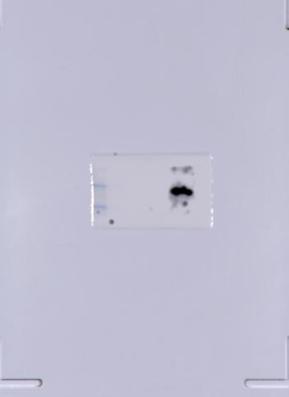


IRF3 p-IRF3 Myc-UL38


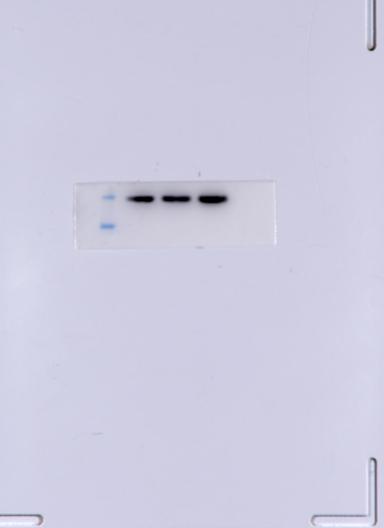


beta-actin

Figure 4

A


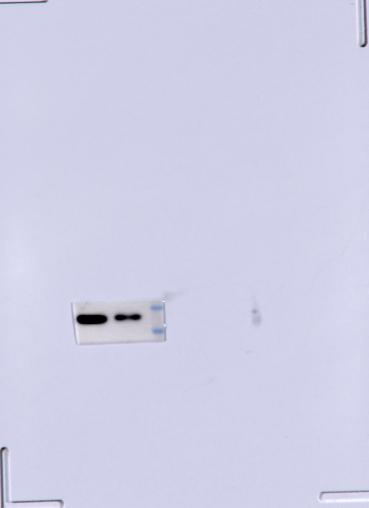

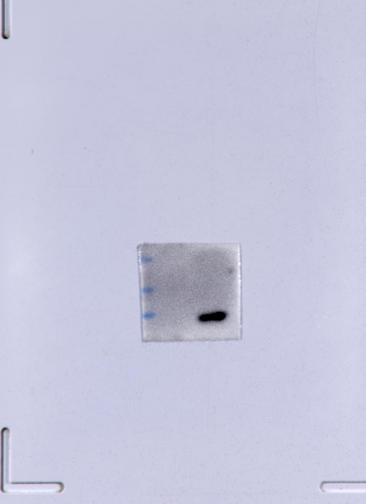

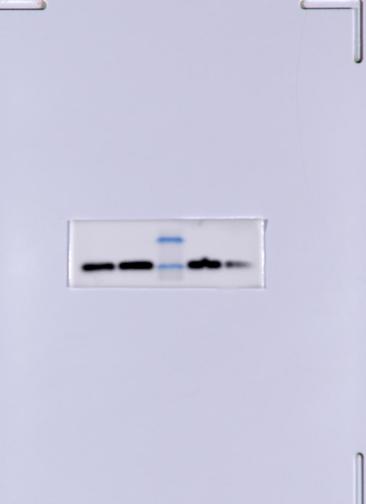


HA Myc beta-actin

B


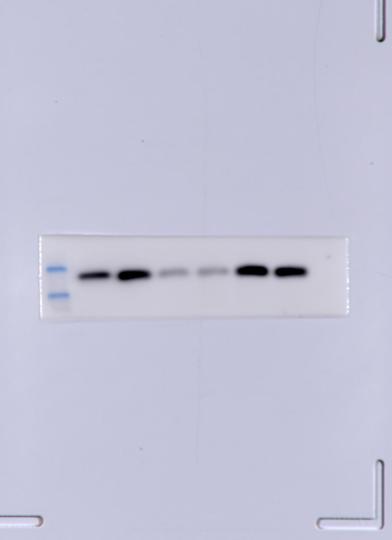

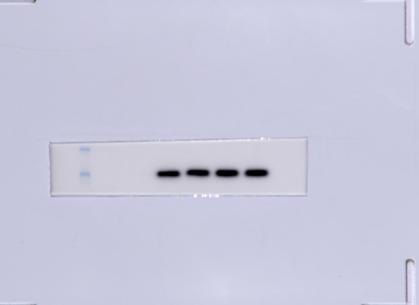

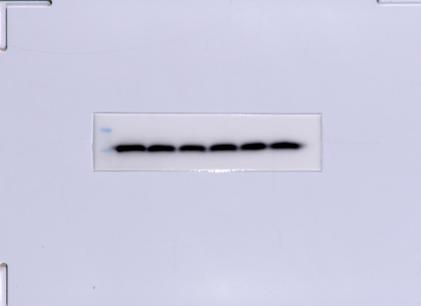


STING Myc-UL38 beta-actin

Figure 5

A


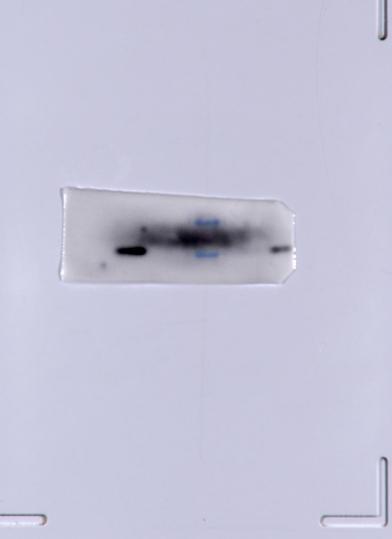

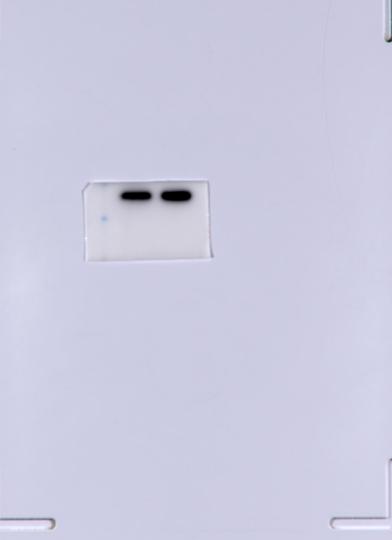


IP-Myc-UL38 IP-HA-STING


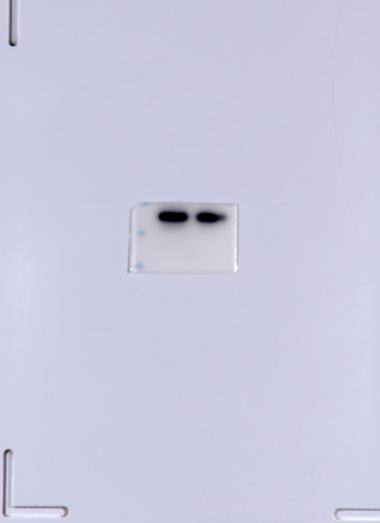

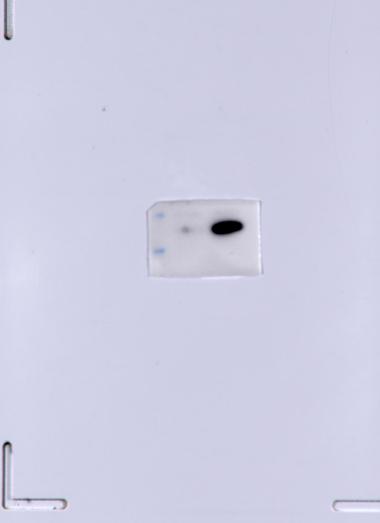

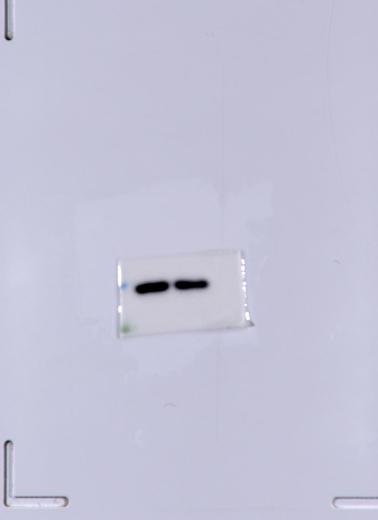


Input-HA-STING Input-Myc-UL38 GAPDH

B


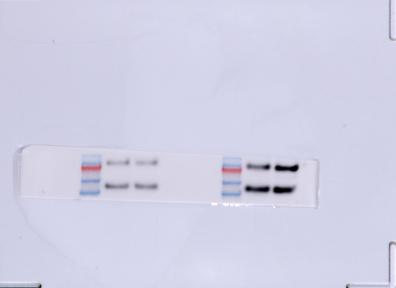

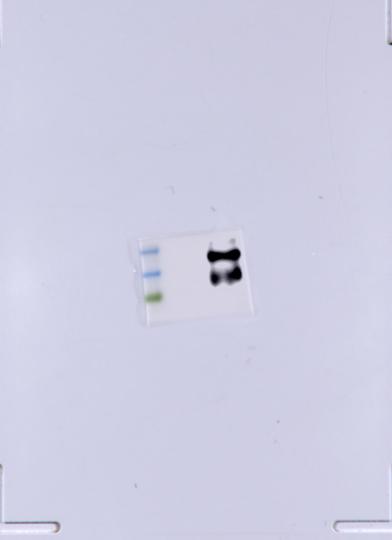


IP-Myc-UL38 IP-HA-STING


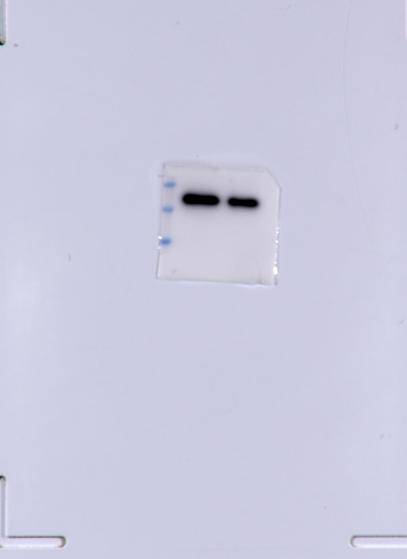

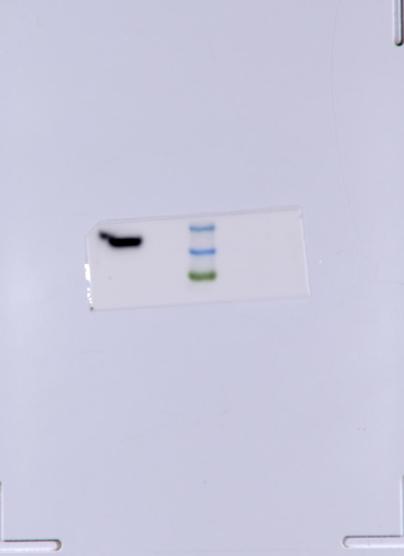

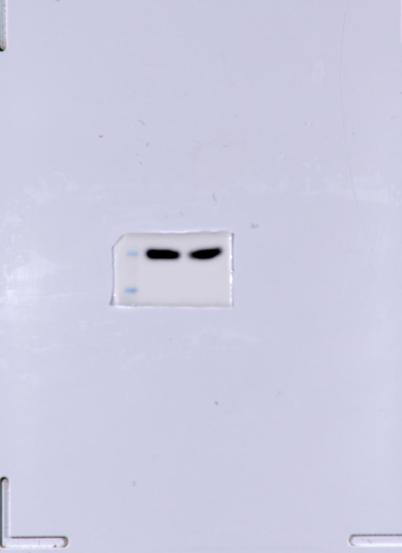


Input-Myc-UL38 Input-HA-STING GAPDH

C


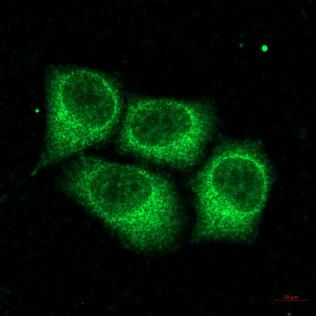

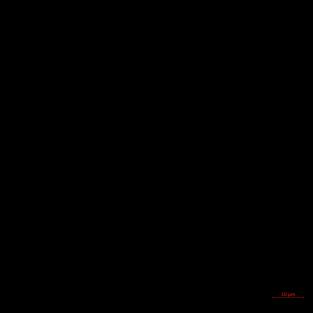

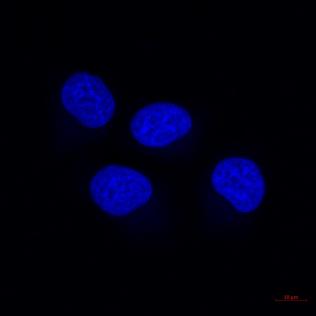


Cy3:HA-STING Coralite488 DAPI


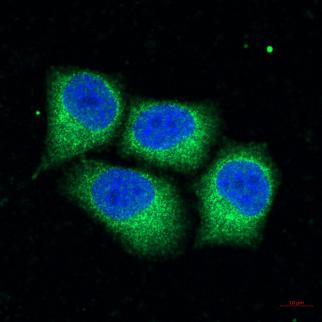


Merge


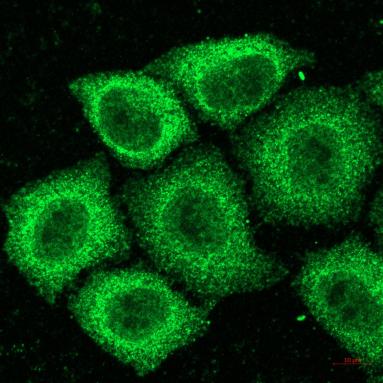

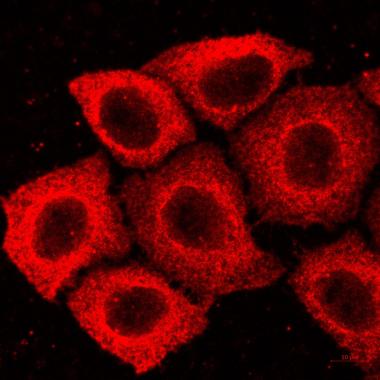

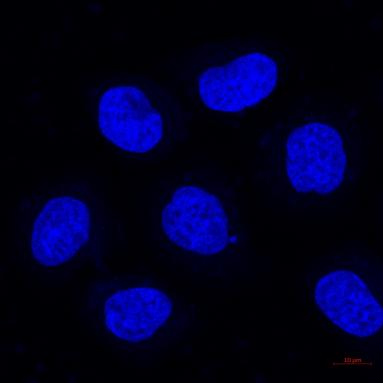


Cy3:HA-STING Coralite488:Myc-UL38 DAPI


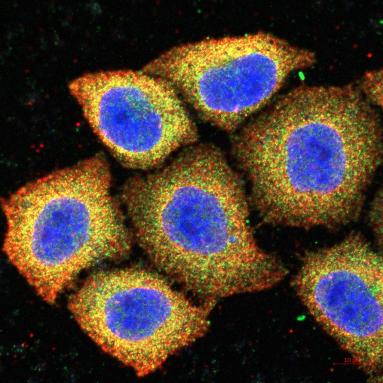


Merge

D


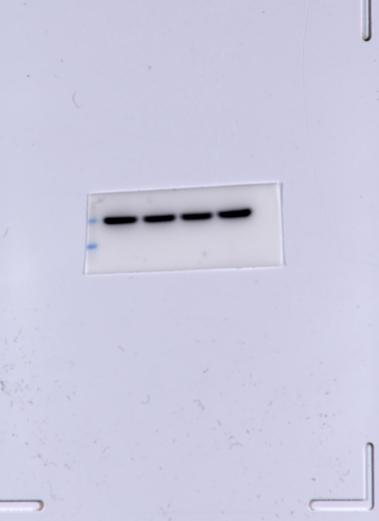

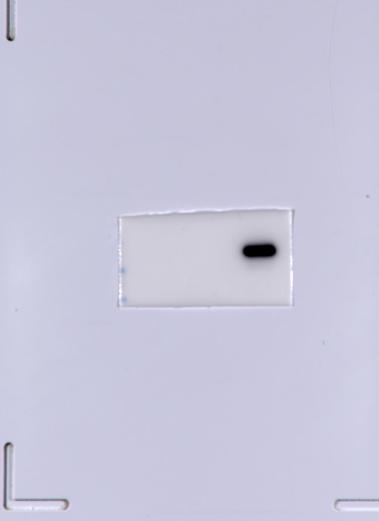

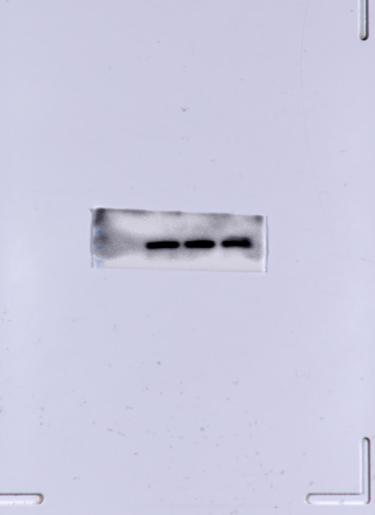


beta-actin Input:FLAG-UL38 Input:HA-STING


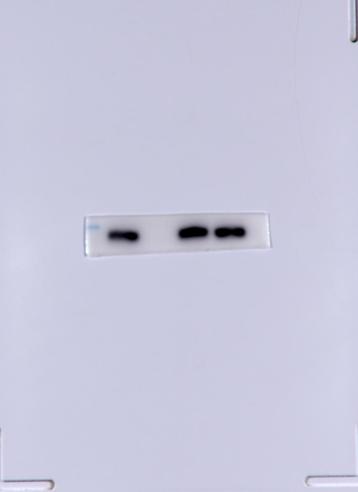


Input:Myc-STING


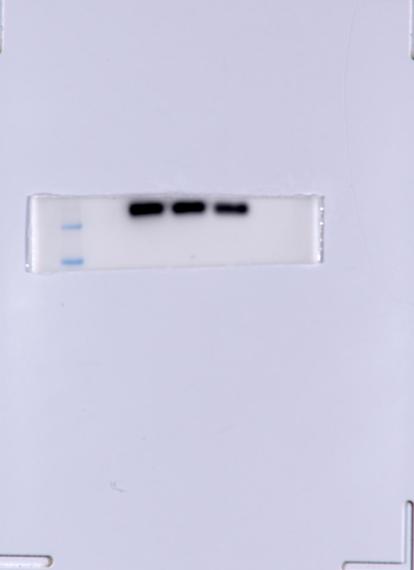

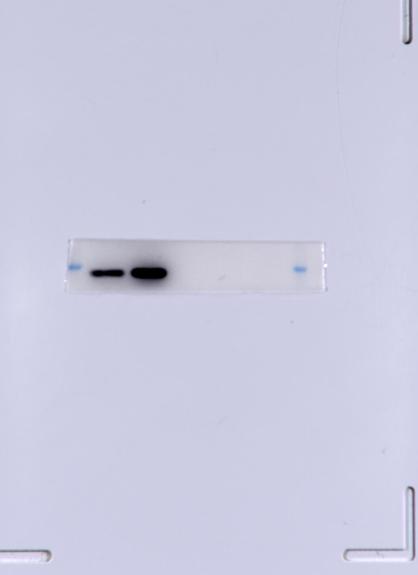

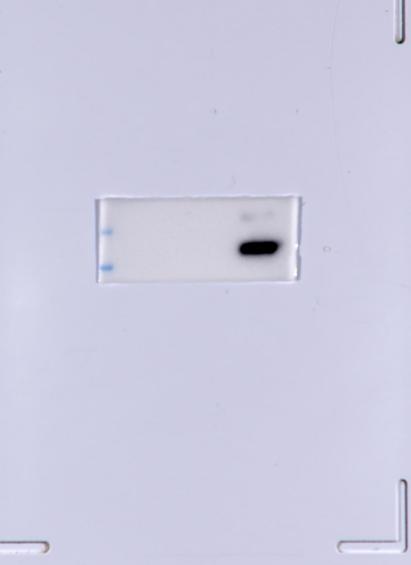


IP:HA-STING IP:Myc-STING IP:FLAG-UL38

E:


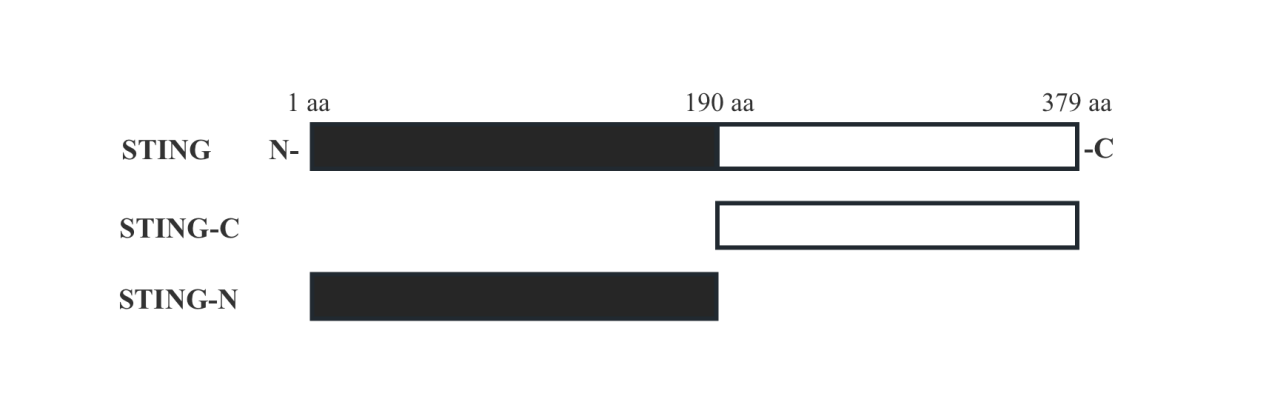


F:


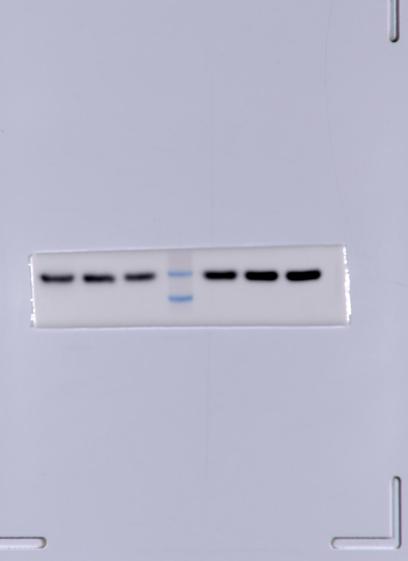

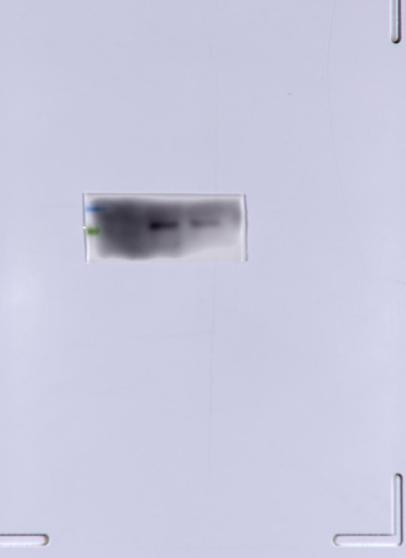

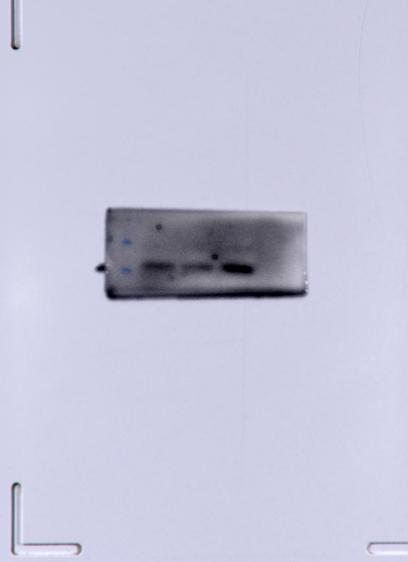


beta-actin Input:HA-STING(C/N) Input-Myc-UL38


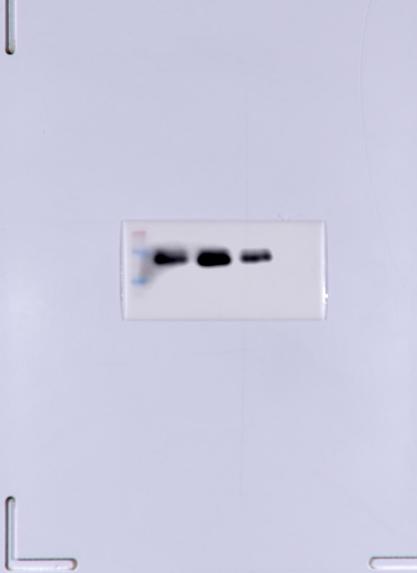

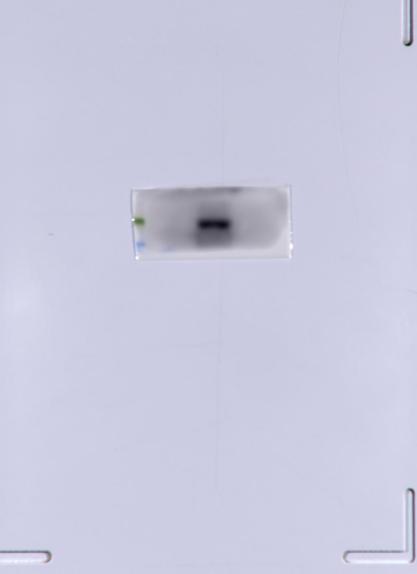


IP-Myc-UL38 IP-HA-STING(C/N)

Figure6

A:


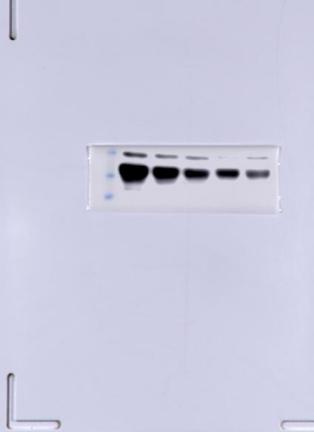


Input: Flag-UL38


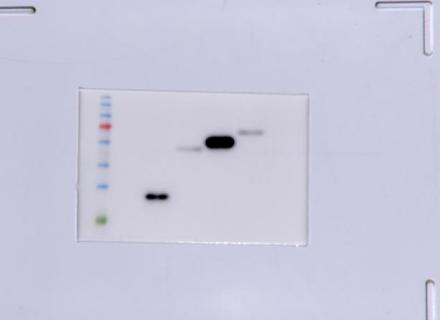

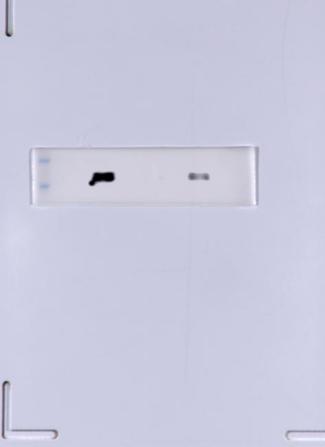


IP: Myc IP:Flag-UL38

B:


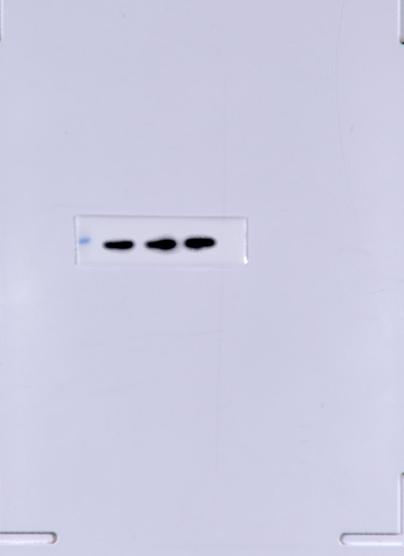

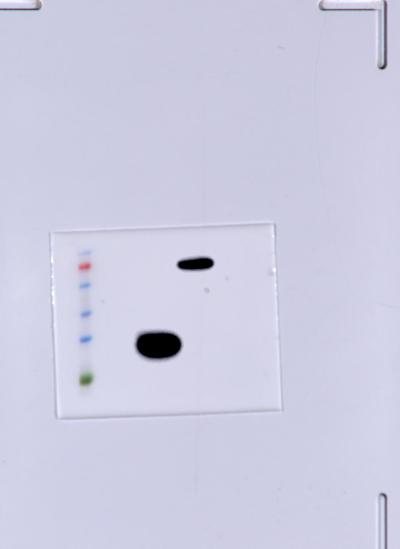

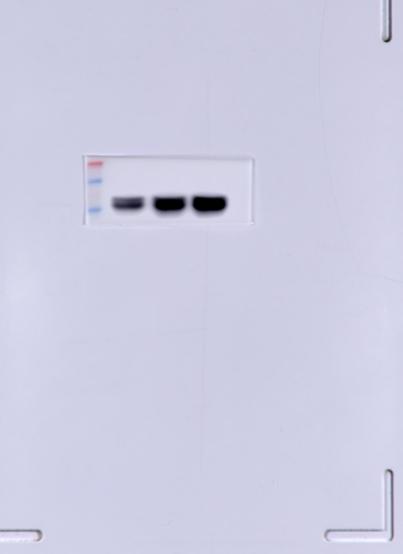


GAPDH Input:Myc Input:Flag-UL38


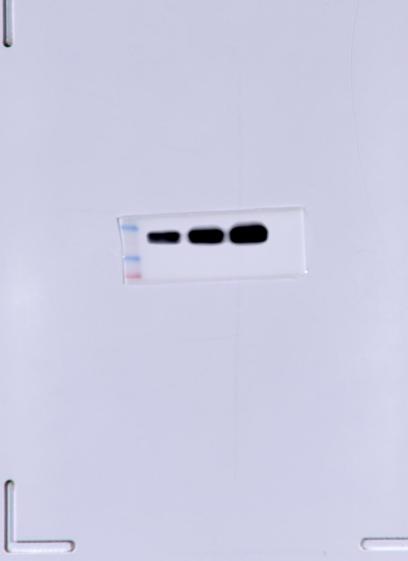

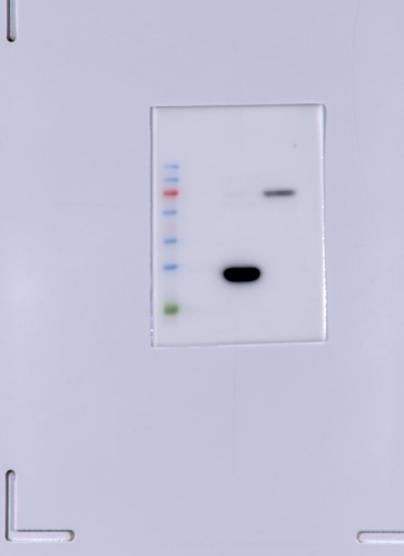


IP:Flag-UL38 IP:Myc

C:


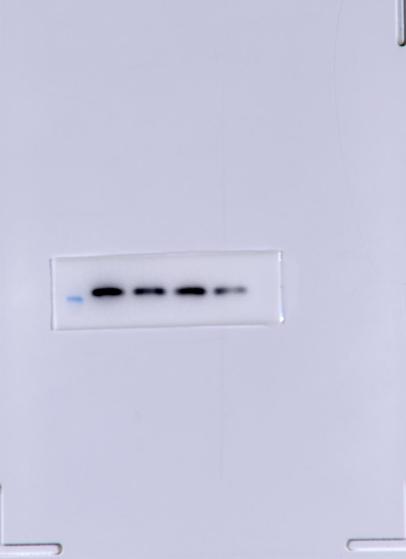

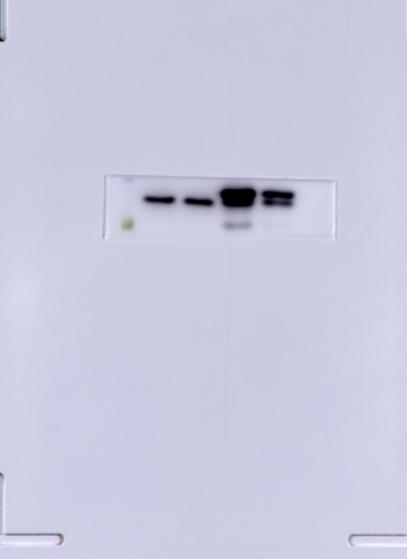

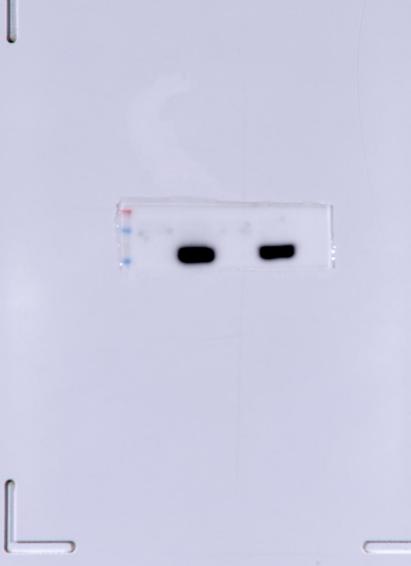


STING Tollip Flag-UL38


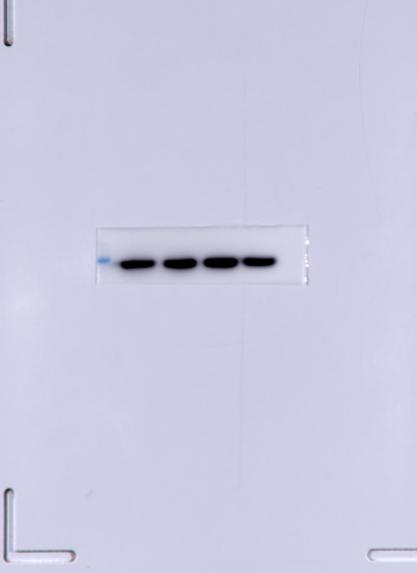


GAPDH

D:


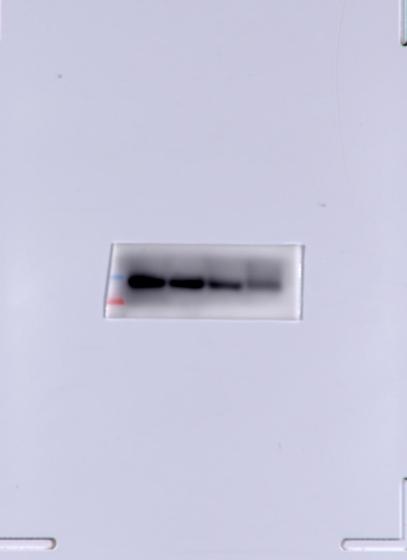

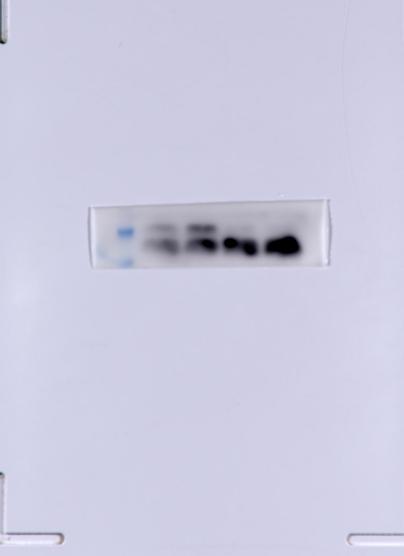

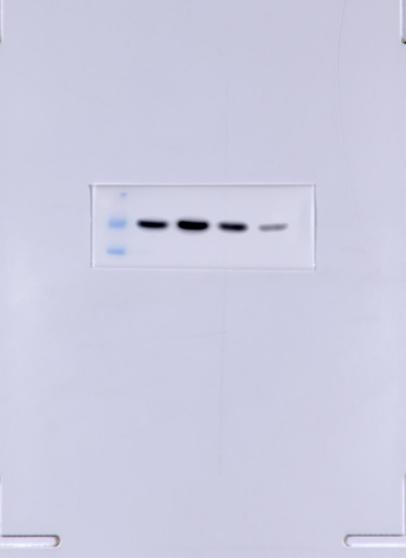


p62 LC-3 Myc-Tollip


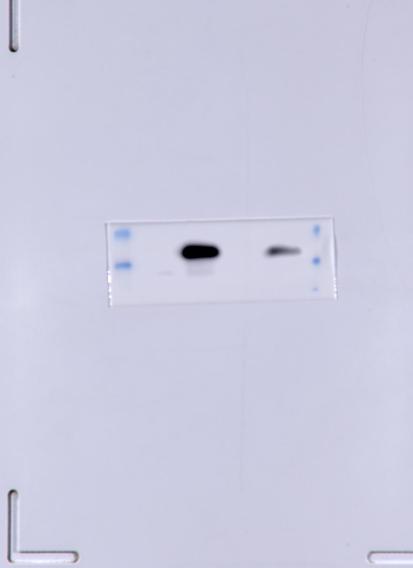

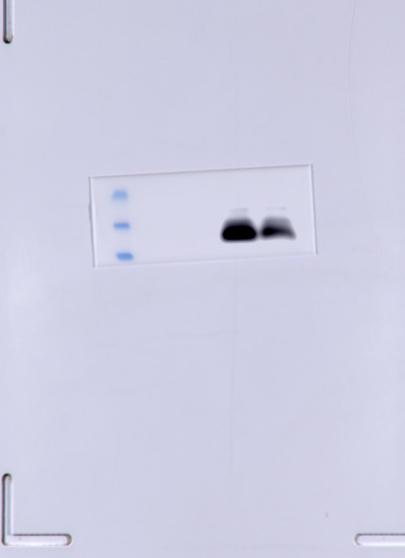

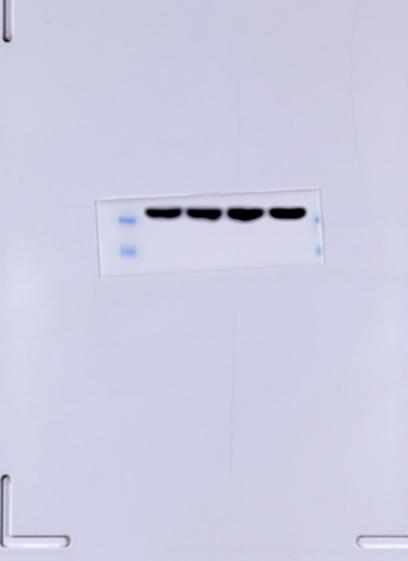


HA-STING Flag-UL38 beta-actin

E:


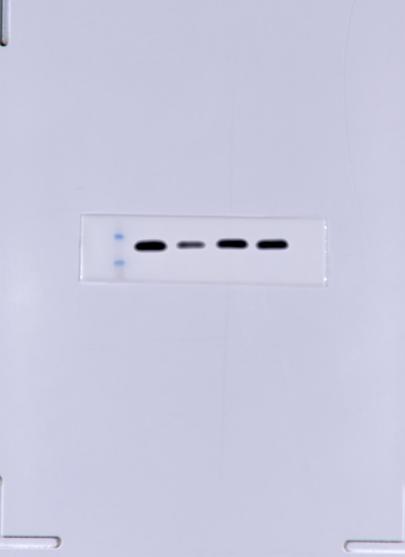

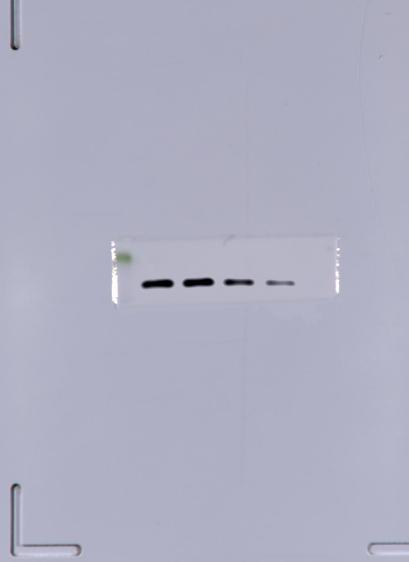

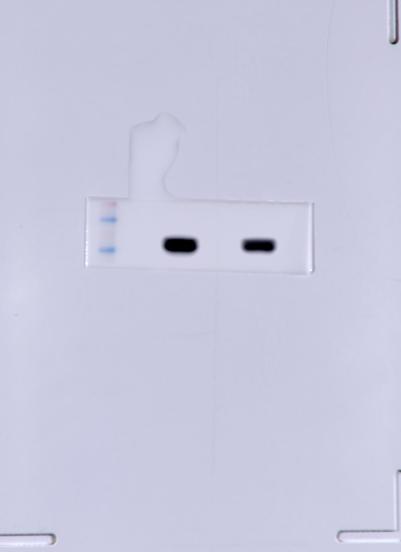


STING Tollip Flag-UL38


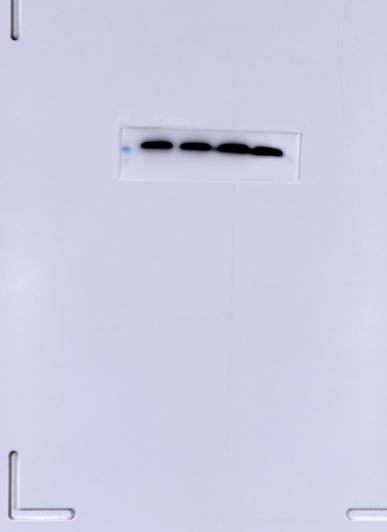


GAPDH

Figure7:

A:

|  | EV+PRV | | UL38+PRV | |
| --- | --- | --- | --- | --- |
| 12 hpi | 2.4 | 2.2 | 3.4 | 3.2 |
| 24 hpi | 3 | 3.2 | 4 | 3.8 |
| 36 hpi | 3.4 | 4.2 | 4.8 | 5.4 |

B:

|  | EV+PRV | | | UL38+PRV | | |
| --- | --- | --- | --- | --- | --- | --- |
| 12 hpi | 3.21870016e+008 | 2.89880704e+008 | 3.42658464e+008 | 7.82251392e+008 | 8.17422464e+008 | 7.82251392e+008 |
| 24 hpi | 5.548392448e+009 | 5.8985088e+009 | 5.63535872e+009 | 1.0444870656e+010 | 6.089358848e+009 | 6.0778368e+009 |
| 36 hpi | 8.815439872e+009 | 8.19960832e+009 | 8.584889856e+009 | 8.591176704e+009 | 8.10254592e+009 | 1.3258596352e+010 |

C:


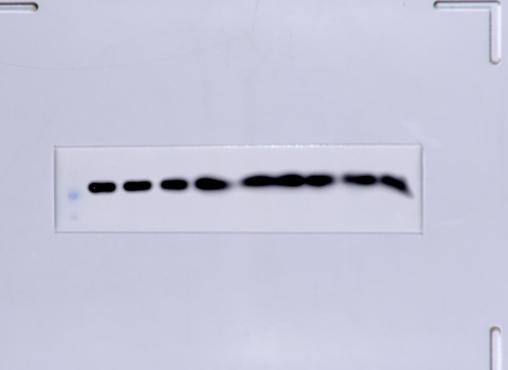

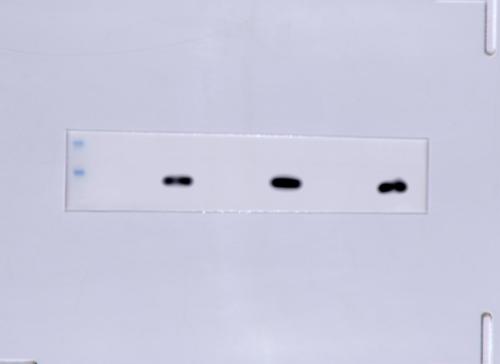


beta-actin Myc-UL38


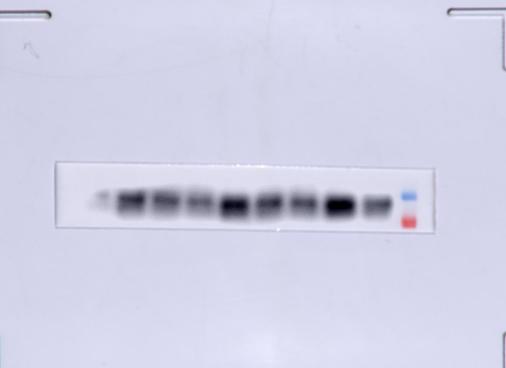

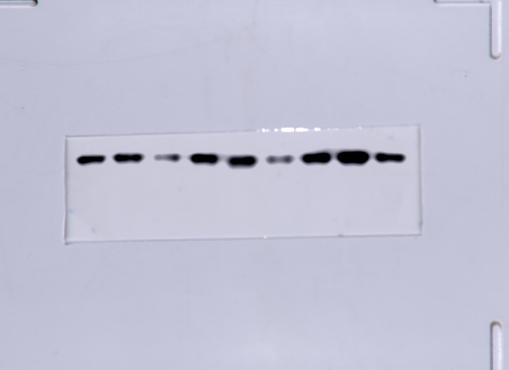


p-TBK1 STING

Figure8:


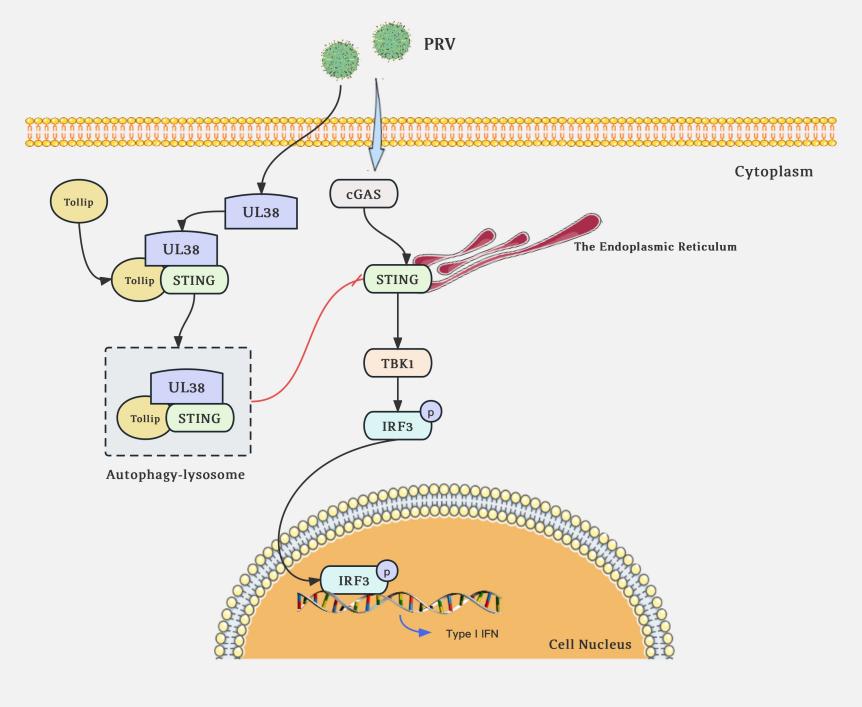


Note：

1. We did RT-qPCR in an ABI 7500 Real-Time PCR system, results exported as Excel format. For ease of reference, I copied the data needed for this study into this document.

2. As we have other studies in our project group, sometimes we do WB experiments at the same time to save time and cost.
